# Thermodynamic description of Eu(NO_3_)_3_-NaNO_3_-H_2_O and Eu(NO_3_)_3_-Mg(NO_3_)_2_-H_2_O systems. Solubility experiments and full dissociation Pitzer models

**DOI:** 10.1039/d5ra09321j

**Published:** 2026-05-20

**Authors:** F. Häusler, P. F. dos Santos, A. Lassin, X. Gaona, K. Garbev, S. Touzelet, Y. Cartigny, M. Altmaier, B. Madé

**Affiliations:** a Institute for Nuclear Waste Disposal, Karlsruhe Institute of Technology Karlsruhe Germany felix.haeusler@kit.edu a.lassin@brgm.fr; b BRGM F-45060 Orléans France; c Institute for Technical Chemistry, Karlsruhe Institute of Technology Karlsruhe Germany; d SMS, Univ. Rouen Normandie F-76821 Mont Saint Aignan France; e Scientific and Technical Division Andra Châtenay-Malabry France

## Abstract

Solubility studies in the systems Eu(NO_3_)_3_-NaNO_3_-H_2_O and Eu(NO_3_)_3_-Mg(NO_3_)_2_-H_2_O were conducted at *T* = (22 ± 2) °C including solid phase identification by X-ray powder diffraction and Schreinemakers' method. Iso-water activity data of both systems at 25 °C were determined by iso-water activity (IWA) and dynamic vapor sorption (DVS) experiments. These experimental results were collectively used to verify and complement existing sets of Pitzer interaction parameters for the ternary systems. The resulting thermodynamic models assume the full dissociation of all involved salts. The geochemical code PhreeSCALE was used together with the parameter estimation software PEST to determine new ternary interaction parameters *Ψ*_Eu^3+^,Na^+^,NO_3_^−^_, and *Ψ*_Eu^3+^,Mg^2+^,NO_3_^−^_. In combination with our previous work on Na_2_SO_4_ and MgSO_4_ solutions [F. dos Santos *et al.*, 2024, *Dalton Trans.*, 53, 6289–6299], the updated model allows an accurate description of Eu(iii) solubility in complex nitrate–sulphate systems of relevance for radioactive waste disposal.

## Abstract 2

Experimental and model phase diagrams for Eu(NO_3_)_3_-NaNO_3_-H_2_O and Eu(NO_3_)_3_-Mg(NO_3_)_2_-H_2_O systems at 25 °C, based on solubility experiments, XRD characterization, Schreinemakers' method, controlled relative humidity experiments and Pitzer equations.

## Graphical abstract

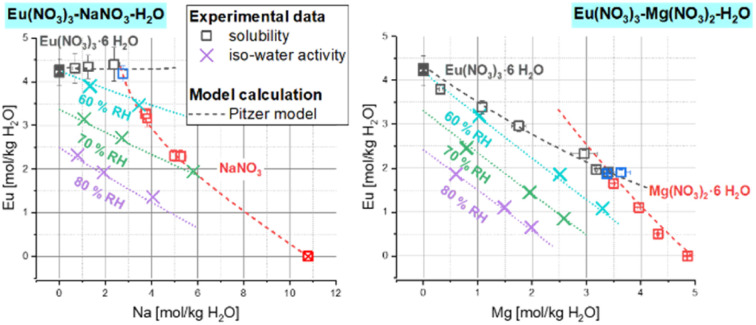

## Introduction

The rare-earth element europium is well known for its luminescence, which has led to versatile applications in light and display technology, security inks, biomedical analysis, and agriculture.^1,2^ As part of the lanthanide series, Eu(iii) is, due to its similar solution chemistry and aqueous speciation, also considered an analogue for trivalent actinides such as Am(iii) and Pu(iii) and is therefore of importance for research in the field of radioactive waste disposal, where both actinides play a predominant role.^2,3^

Considerable quantities of nitrate are part of the disposal inventory in several streams of long-lived intermediate-level and long-lived low-level radioactive waste.^4,5^ In particular, reprocessing techniques using nitric acid to recover radionuclides result in significant inventories of nitrate-rich waste.^6–9^ Nitrate is known to be an oxidizing agent and therefore able to influence the redox chemistry within a nuclear waste repository.^10,11^ A comprehensive understanding of the solution chemistry of nitrate systems is also required to predict interactions with (geo-)technical barriers and the impact on radionuclide retention.

Aqueous nitrate systems are known for high ionic strength due to the comparatively high solubility of nitrate salts.^12–16^ To properly describe the chemical behaviour of such systems, it is thus important to collect sufficient experimental data over a wide concentration range and use models able to reproduce solution properties and solubility behaviour at high ionic strength. The Pitzer model^17,18^ fulfils this requirement and is therefore used in the present work. Following the full dissociation approach and taking advantage of specific interaction parameters previously published for the binary systems, sets of Pitzer parameters are proposed to properly describe the two ternary systems Eu(NO_3_)_3_-NaNO_3_-H_2_O and Eu(NO_3_)_3_-Mg(NO_3_)_2_-H_2_O. New investigations on solubility in diluted to concentrated aqueous solutions (up to 5.3 mol NaNO_3_ per kg H_2_O and 4.3 mol Mg(NO_3_)_2_ per kg H_2_O) and iso-water activity experiments were conducted as the data basis for the parameter estimation process. Future work will focus separately upon the alternative partial dissociation approach, which considers the formation of aqueous metal–nitrate complexes and includes SIT (specific ion interaction theory) and Pitzer models as well as complementary spectroscopic data on Eu(iii)-NO_3_^−^ speciation.

## Experimental

### Chemicals

Solutions were prepared with ultrapure water, purified with a Milli-Q Academic apparatus (Merck Millipore, 18.2 MΩ cm, 22 ± 2 °C, pore size: 0.22 µm). Anhydrous sodium nitrate (NaNO_3_, p.a., 99 wt%), magnesium nitrate hexahydrate (Mg(NO_3_)_2_·6H_2_O, p.a., 99 wt%), europium(iii) nitrate hexahydrate (Eu(NO_3_)_3_·6H_2_O, p.a., 99.9 wt%) and europium(iii) sulphate octahydrate (Eu_2_(SO_4_)_3_·8H_2_O, p.a., 99.9 wt%) were purchased from Thermo Fisher Scientific. Anhydrous sodium sulphate (Na_2_SO_4_, p.a., 99 wt%) was purchased from Merck.

### Solubility experiments

Solubility studies were performed with different series of independent batch experiments from undersaturation conditions. Nine and twelve samples for the systems Eu(NO_3_)_3_-NaNO_3_-H_2_O and Eu(NO_3_)_3_-Mg(NO_3_)_2_-H_2_O were equilibrated at *T* = (22 ± 2) °C over concentration ranges of 0.0–5.3 mol NaNO_3_ per kg H_2_O and 0.0–4.3 mol Mg(NO_3_)_2_ per kg H_2_O, respectively. The pH values of these samples were determined with an Orion 8103SC pH glass electrode (Thermo Scientific) after calibration with three commercial calibration buffer solutions of pH = 1, 4, and 7. An additional set of six samples was prepared for the quaternary system Eu_2_(SO_4_)_3_-Eu(NO_3_)_3_-Na_2_SO_4_-NaNO_3_-H_2_O with 1.3–4.5 mol NaNO_3_ per kg H_2_O and 0.8–1.6 mol Na_2_SO_4_ per kg H_2_O. Experiments in pure nitrate systems were conducted from undersaturation conditions with Eu(NO_3_)_3_·6H_2_O, whereas Eu_2_(SO_4_)_3_·8H_2_O was used for solubility in mixed nitrate–sulphate solutions. All samples were subjected to constant agitation until thermodynamic equilibrium was reached, which was assumed after repeated measurements showing constant total Eu(iii) concentration. Sampling was performed several times over a period of up to 21 months. Solid and liquid phases were separated by ultrafiltration (10 kDa ≈ 2 nm, Pall Life Science), followed by the dilution of solutions with HNO_3_ (2%). A dilution factor between 10 000 and 50 000 was applied for analysis due to the high salt concentrations. Eu, Na, Mg, and S concentrations were then measured *via* ICP-OES (Inductively Coupled Plasma Optical Emission Spectroscopy, PerkinElmer Optima 8300 DV). The uncertainty of ICP-OES measurements is below 5%. Uncertainties for solubility data depicted in the related figures are calculated as standard deviations of the average of single measurements for each sample (>15 measurements per sample).

### Iso-water activity (IWA) and dynamic vapor sorption (DVS) experiments

To add constraints to specific ion interaction optimization, experiments were carried out to measure the chemical composition of synthetic brines at equilibrium with fixed relative humidities at 25 °C. The principle is the following: an aqueous mixture of known composition and mass is placed in an environment of fixed relative humidity and temperature. Its mass is monitored continuously over several hours to days until it stabilizes, which is representative of thermodynamic equilibrium. The mass variation is due to water gain or loss, and its final value therefore indicates the chemical composition of the brine at equilibrium with the stipulated relative humidity, equivalent of the water activity. This iso-water activity (IWA) technique is similar to isopiestic measurements,^20^ with the difference that water activity is imposed by means of controlled relative humidity.^9,21,22^

Two different apparatuses were used. The first one is a climatic chamber C-20/350 (CTS GmbH), whose internal volume is 350 L. Temperature and relative humidity can be varied in the ranges −20–180 °C, and 10–98%, respectively. The uncertainty of the measured temperature and relative humidity is 0.03 °C and 1.5%, respectively. A beaker of 100 mL was placed in the climatic chamber on a balance (Combics 3, Sartorius Weighing Technology GmbH, precision of 0.01 g) and the brine was continuously stirred with a paddle. The initial mass of the salt solution was *ca.* 50 g. At the end of the experiment for a given relative humidity, the weight was measured without the paddle to avoid Archimedes' buoyancy. Then, a new value of the relative humidity was set for a new experiment. The chamber needs to be opened for 1–2 minutes to adjust the paddle. The following stabilization of temperature and relative humidity takes about one hour. The frequency of measurements was once per day to ensure sufficient equilibration time and minimization of the error in weight measurement.

The second apparatus is a dynamic vapor sorption (DVS) device DVS One (Surface Measurement Systems, Wembley, UK). In this apparatus, the temperature (±0.5 °C) and the relative humidity (±0.5%) are regulated, while the mass variation is recorded by a microbalance (±0.1 µg). Samples were submitted to the successive desorption–sorption cycles (80–50% relative humidity with a step size of 10% RH). The fixed criterion for the step change was a mass variation of the sample lower than 0.0005% per minute in the limit of 2000 min per step. The DVS device is composed of a thermostatic chamber with two closed vessels; each contains a suspended cup connected to a balance. A drop (*i.e.* less than 0.1 g) of the brine to study was placed in one cup, while the second cup remained empty as reference. Like in the climatic chamber, the relative humidity is set in the air that flows independently through the two vessels. Here again, the relative mass variation indicates the variation of the chemical composition of the brine due to water evaporation or condensation. The mass is continuously measured without the need to open the chamber. Because of the much smaller masses of brine used with the DVS device, experiments reach equilibrium faster than in the climatic chamber.

For both methods the chambers rely on the mixture of humid and dry air to set the targeted relative humidity, which is a method not expected to impact the equilibria.

### X-ray powder diffraction (XRPD)

Solid phases were identified by X-ray powder diffraction. Measurements of samples A–J (with sample A being the solid from the Eu(NO_3_)_3_-H_2_O system, B–F are samples of the Eu(NO_3_)_3_-NaNO_3_-H_2_O system and G–J of the Eu(NO_3_)_3_-Mg(NO_3_)_2_-H_2_O system; see Table 1 for detailed description) were performed in modified Si sample holders with cavity (1 mm depth) with an Empyrean diffractometer (Malvern Panalytical) using Cu-K_α_ radiation. A Bragg–Brentano HD device (Malvern Panalytical) was used to isolate the K_α_ from the K_β_ radiation and to reduce the fluorescence and the background. The primary optics included divergent slits (1/16°), antiscattering slits (1/4°), mask 4 mm and soller slits (0.04 Rad). Detection was conducted by a multi-strip PIXcel 3D detector covering 3.348 °2*θ* simultaneously with 255 channels. The scan range was 5–90 °2*θ* with a 0.013 °2*θ* step size and 3.14 s per step.

Solubility data and solid phase characterization of this work for the systems Eu(NO_3_)_3_-H_2_O, Eu(NO_3_)_3_-NaNO_3_-H_2_O and Eu(NO_3_)_3_-Mg(NO_3_)_2_-H_2_O at (22 ± 2) °C and pH ≤ 3.8

**Table:** 

Eu(NO_3_)_3_-H_2_O					
m(Eu(NO_3_)_3_) in mol per kg H_2_O	XRPD sample	Solid phase^aa^	Rietveld refinement results in wt% (e.s.d.)		
			Eu(NO_3_)_3_·6H_2_O	Eu(NO_3_)_3_(H_2_O)_4_(H_2_O)	NaNO_3_
4.22 ± 0.34	A	Eu(NO_3_)_3_·6H_2_O	70(1)	30(1)	—

a Identification of solid phases: XRPD.

b Schreinemakers' method.

**Table:** 

Eu(NO_3_)_3_-NaNO_3_-H_2_O						
m(NaNO_3_) in mol per kg H_2_O	m(Eu(NO_3_)_3_) in mol per kg H_2_O	XRPD sample	Solid phase^a^			
0.68 ± 0.12	4.31 ± 0.35	B	Eu(NO_3_)_3_·6H_2_O	90(1)	10(1)	—
1.24 ± 0.19	4.34 ± 0.27	C	Eu(NO_3_)_3_·6H_2_O	81(4)	19(4)	—
2.37 ± 0.24	4.40 ± 0.39					
2.75 ± 0.21	4.18 ± 0.18	D	Eu(NO_3_)_3_·6H_2_O + NaNO_3_	71(2)	12(1)	17(2)
3.73 ± 0.21	3.27 ± 0.11					
3.81 ± 0.16	3.16 ± 0.19	E	NaNO_3_	—	—	100
5.26 ± 0.24	2.29 ± 0.14					
5.00 ± 0.22	2.30 ± 0.14	F	NaNO_3_	—	—	100

**Table:** 

Eu(NO_3_)_3_-Mg(NO_3_)_2_-H_2_O						
m(Mg(NO_3_)_2_) in mol per kg H_2_O	m(Eu(NO_3_)_3_) in mol per kg H_2_O	XRPD sample	Solid phase^a^^,^^b^	Rietveld refinement results in wt% (e.s.d.)		
				Eu(NO_3_)_3_·6H_2_O	Eu(NO_3_)_3_(H_2_O)_4_(H_2_O)	Mg(NO_3_)_2_·6H_2_O
0.31 ± 0.03	3.80 ± 0.11	G	Eu(NO_3_)_3_·6H_2_O	97(1)	3(1)	—
1.09 ± 0.08	3.38 ± 0.15	H	Eu(NO_3_)_3_·6H_2_O	97(1)	3(1)	—
1.75 ± 0.13	2.96 ± 0.12	I	Eu(NO_3_)_3_·6H_2_O	80(3)	20(3)	—
2.96 ± 0.24	2.33 ± 0.08	J	Eu(NO_3_)_3_·6H_2_O	59(3)	41(3)	—

**Table:** 

Eu(NO_3_)_3_-Mg(NO_3_)_2_-H_2_O			
m(Mg(NO_3_)_2_) in mol per kg H_2_O	m(Eu(NO_3_)_3_) in mol per kg H_2_O	XRPD sample	Solid phase^a^^,^^b^
3.63 ± 0.17	1.90 ± 0.06	K	2Eu(NO_3_)_3_·3Mg(NO_3_)_2_·24H_2_O + Mg(NO_3_)_2_·6H_2_O
3.18 ± 0.01	1.97 ± 0.02	L	Eu(NO_3_)_3_·6H_2_O
3.40 ± 0.01	1.91 ± 0.02	M	Eu(NO_3_)_3_·6H_2_O
3.38 ± 0.03	1.88 ± 0.01	N	Eu(NO_3_)_3_·6H_2_O + Mg(NO_3_)_2_·6H_2_O
4.31 ± 0.02	0.51 ± 0.01	O	Mg(NO_3_)_2_·6H_2_O
3.96 ± 0.03	1.11 ± 0.01	P	Mg(NO_3_)_2_·6H_2_O
3.50 ± 0.03	1.65 ± 0.01	Q	Mg(NO_3_)_2_·6H_2_O
3.38 ± 0.03	1.86 ± 0.01	R	Eu(NO_3_)_3_·6H_2_O + Mg(NO_3_)_2_·6H_2_O

Measurements of samples K–R (all samples of the Eu(NO_3_)_3_-Mg(NO_3_)_2_-H_2_O system; see Table 1) were performed with a diffractometer D8 Advance (Bruker AXS) with a Lynxeye XE-T detector (1D mode) in Bragg–Brentano set-up using Cu-K_α_ radiation (the high energy resolution of 380 eV of the detector allows the selection of Cu-K_α_ radiation without an additional monochromator). A step size of 0.013 °2*θ* with 0.1–0.2 s per step was applied within the scan range of 5–60 °2*θ*.

The preparation of samples for measurement was kept as short as possible to prevent phase changes of the hygroscopic samples. A fraction of the solid phase was taken from the sample, roughly dried with absorbent paper and instantly prepared for measurement. Usually, three measurements of a sample were conducted in a row to identify time dependent phase changes and secondary crystallizations. The washing of samples with ethanol was tested, but resulted in high dissolution and subsequent crystallization, leading to a varying phase assemblage, which not necessarily represents equilibrium phases. Therefore, the former method without washing was preferred, despite the known disadvantages. A summary of XRPD measurements is presented in the SI of this work.

### Rietveld refinement

Samples A–J have been evaluated and quantified by Rietveld refinement performed with TOPAS V.7 (Bruker-AXS, Karlsruhe, Germany). The structures of Eu(NO_3_)_3_(H_2_O)_6_ (ICSD 280528) and Eu (NO_3_)_3_(H_2_O)_4_(H_2_O) (ICSD 61257) were used as starting models for samples A–D and G–J.^23,24^ For sample D, the structure of nitratine (NaNO_3_, ICSD 14185) was additionally taken into account.^25^ Samples E and F were refined using solely the structure of nitratine. The profile fitting was performed with the fundamental parameters approach (TOPAS V.7 Technical reference, Bruker-AXS) in the range 5–80 °2*θ*. The size of the coherent scattering domains was calculated by the double Voigt approach^26^ implemented in TOPAS V.7.

### Schreinemakers' method

Schreinemakers' method^27^ was executed for the Eu(NO_3_)_3_-Mg(NO_3_)_2_-H_2_O samples to verify phase compositions despite fast hygroscopicity effects of solid phases outside their mother liquor, which were observed during XRPD measurements. Fractions of each sample with varying liquid/solid ratios were weighted (79–281 mg) and afterwards dissolved in 10 mL 2% HNO_3_. The resulting solutions were further diluted (1 : 250–1 : 500), followed by the analysis of Eu and Mg contents *via* ICP-OES as described earlier for the solubility experiments. Resulting compositions of suspensions and solutions were converted to mass fractions of Eu(NO_3_)_3_, Mg(NO_3_)_2_, and H_2_O (the water content was calculated on the basis of Eu and Mg contents) and plotted in a ternary diagram to deduce the solid phases in equilibrium by extrapolation.

### Thermodynamic modelling

The activities *a*_*i*_ (−) of solvent and solute describe the deviation from ideal behaviour induced by interactions in solution. For solutes, activity coefficients *γ*_*i*_ (−) quantify this deviation by1
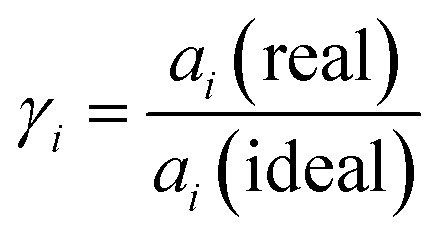
which leads to the correlation of activity and the measurable molality of real solutions:2
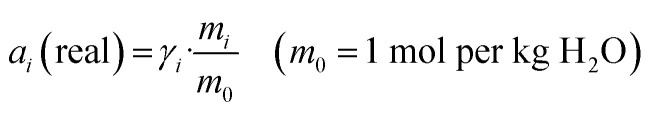


The solubility behaviour of solids is accordingly required to describe solid–liquid equilibria. For dissolution/precipitation processes, the following equation applies:3
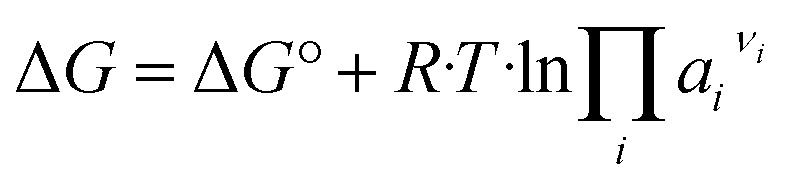
with Δ*G* – free energy change (which results 0 J mol^−1^ in thermodynamic equilibrium), Δ*G*^°^ – standard free energy change, *R* – universal gas constant (8.314 J mol^−1^ K^−1^), *T* – temperature in K and *ν*_*i*_ – stoichiometric coefficient (−) of species *i*. Solids are generally considered as pure phases, with constant unit activity. For thermodynamic equilibrium, the following further applies4
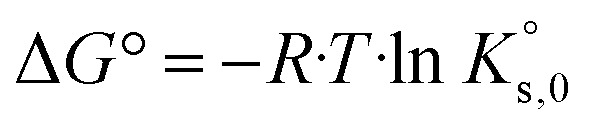
where 
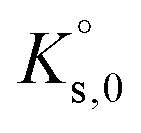
 is the solubility constant (−) of a given solid phase. By combining eqn (3) and (4) one obtains:5
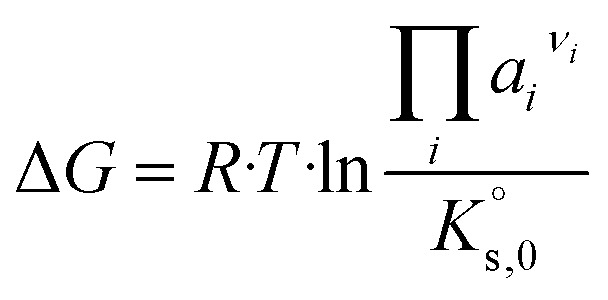
6
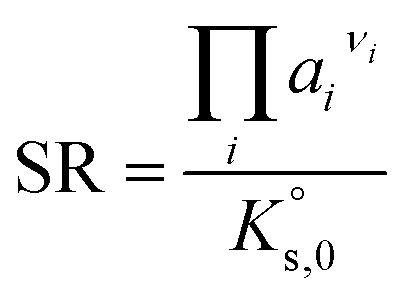


The saturation ratio SR (−), defined as the quotient of ion activity product and solubility constant, equals 1 at equilibrium. For SR > 1 a solution would be supersaturated, while at SR < 1 it is undersaturated with respect to the solid. The saturation ratio is used in the present work to model the solubility curves and compare calculated and experimental values.

The activity of the solvent water, *a*_w_, is also influenced by the interactions caused by dissolving salts. In contrast to activity coefficients of the electrolytes, the change for the activity coefficient of water is comparatively low in moderately concentrated solutions (relative to the electric charge of the solutes, and thus on the ionic strength). Thus, the numerically more sensitive osmotic coefficient *φ* (−) is usually used to quantify those changes according to Dinane:^28^7
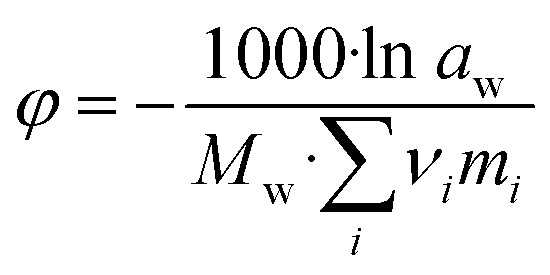
with *a*_w_ – water activity, *M*_w_ – molar mass of water (18.02 g mol^−1^), *m*_*i*_ – molality (mol per kg H_2_O) of dissolved electrolyte *i* and *ν*_*i*_ – the number of ions produced by the total dissociation of electrolyte *i*. Osmotic coefficients are used in the present work to converge proposed models to the experimental results of IWA/DVS experiments.

An essential task in geochemical modelling of saline solutions is the description of ionic interactions, which gain relevance and complexity with increasing ionic strength. A basis for many models is the (extended) Debye–Hückel model, which is able to describe long-range interactions for very dilute solutions (<10^−2^–10^−1^ mol per kg H_2_O).^17,29–31^ Higher ionic strengths demand the introduction of additional parameters to describe the increasing influence of short-range interactions. This is required especially at high molalities, which are certainly expected for nitrate salts.^12–16^ The Pitzer model^17^ is one of the most promising approaches for this purpose and is by now widely used in consistent thermodynamic databases^32,33^ to describe high saline solutions. A brief summary of the Pitzer equations is given in the SI of F. dos Santos *et al.*^34^ The reader is referred to the original Pitzer publications^17,18^ for a more detailed description of these equations.

The present work is based on the full dissociation approach for dissolved salts considering Eu^3+^, Na^+^, Mg^2+^ and NO_3_^−^ as aqueous species. Previous studies^30,35,36^ showed good descriptions of solubility behaviour with the full dissociation assumption. In this way the semi-empirical parameters consider a possible formation of ion pairs or aqueous complexes as specific interactions without explicitly stating their existence. This drastically reduces the number of parameters needed and therefore reduces the present risk of overparameterization.

However, considering ion pairs and aqueous complexes enables a more realistic description of the chemical behaviour. Previous studies state the existence of europium nitrate^37,38^ and sodium nitrate^39^ complexes in solution. MgNO_3_^+^ seems also worth considering based on the description of various other MgX^+^ (X^−^ = F^−^, Cl^−^, Br^−^, I^−^, HCO_3_^−^, …) and ANO_3_^+^ (A^2+^ = Ca^2+^, Sr^2+^, Ba^2+^, Mn^2+^, Ni^2+^, …) aqueous species in the ThermoChimie^40^ database (and references therein). The inclusion of complexes in SIT and Pitzer partial dissociation models is the main subject of future work.

### Parametrization procedure

Modelling the solubility behaviour of aqueous ternary systems requires a consistent set of parameters, which, for a full dissociation Pitzer approach, includes the decadic logarithm of solubility constants at infinite dilution 
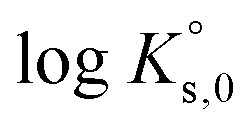
 of involved solids as well as specific binary (*β*^(0)^_*i*,*j*_, *β*^(1)^_*i*,*j*_, *β*^(2)^_*i*,*j*_, *C*_*i*,*j*_^*ϕ*^) and ternary (*θ*_*i*,*k*_, *Ψ*_*i*,*k*,*j*_) Pitzer interaction parameters (where *i* and *k* designate cations and *j* represents an anion).

The parametrization procedure is based on experimental solubility, DVS, and IWA data of the ternary systems Eu(NO_3_)_3_-NaNO_3_-H_2_O and Eu(NO_3_)_3_-Mg(NO_3_)_2_-H_2_O (obtained in the present work) and interaction parameters for the binary subsystems Eu(NO_3_)_3_-H_2_O, NaNO_3_-H_2_O, and Mg(NO_3_)_2_-H_2_O, which were determined by Guignot *et al.*^41^ and Lach *et al.*^42^ In a first step, the binary parameters were tested within their respective systems against experimental literature data^43–49^ and kept constant for the ternary systems to ensure consistency within the database. Additional needed cation interaction parameters *θ*_Eu^3+^,Na^+^_ and *θ*_Eu^3+^,Mg^2+^_ were taken from the work of F. dos Santos *et al.*^34^ on the respective sulphate systems, while ternary parameters *Ψ*_Eu^3+^,Na^+^,NO_3_^−^_, and *Ψ*_Eu^3+^,Mg^2+^,NO_3_^−^_ were determined in the present work. To do so, the parameter estimation software PEST^50^ was coupled with the geochemical calculation code PhreeSCALE^51^ and its corresponding database^32^ as earlier described in F. dos Santos *et al.*^34^ New ternary parameters were estimated based on 70 data points from solubility and iso-water activity experiments of this work.

The optimization procedure aims at minimizing the deviation between the resulting models and the experimental data, which is described with sigma values, *σ*, as utilized in the work of Christov & Moller:^52^8
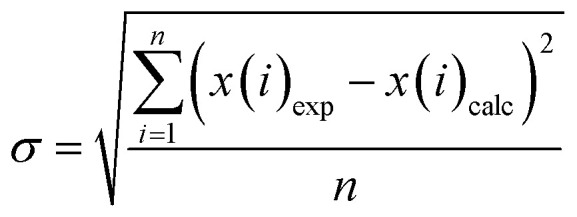
with the experimental and calculated values *x*(*i*)_exp_ and *x*(*i*)_calc_ for a data point *i* and *n* as the number of data points in the respective set. For solubility data (9) and osmotic coefficients (10) this leads to:9
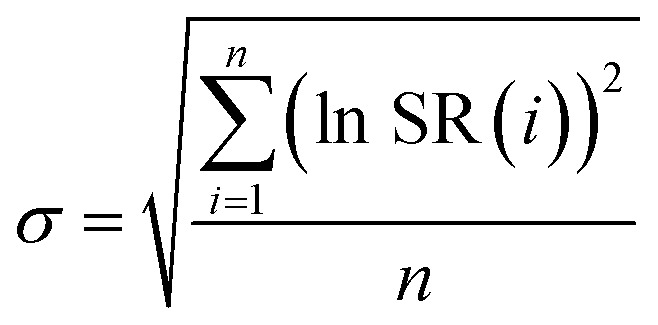
10
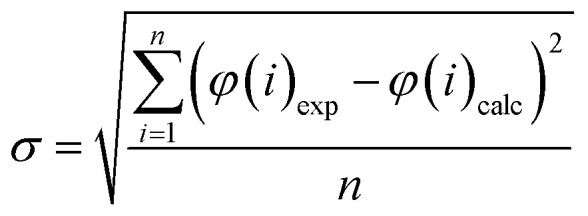


## Results and discussion

### Solubility and iso-water activity data

The determined solubility data of the systems Eu(NO_3_)_3_-H_2_O, Eu(NO_3_)_3_-NaNO_3_-H_2_O and Eu(NO_3_)_3_-Mg(NO_3_)_2_-H_2_O at *T* = (22 ± 2) °C and pH ≤ 3.8 are listed in Table 1.

**Fig. 1:**
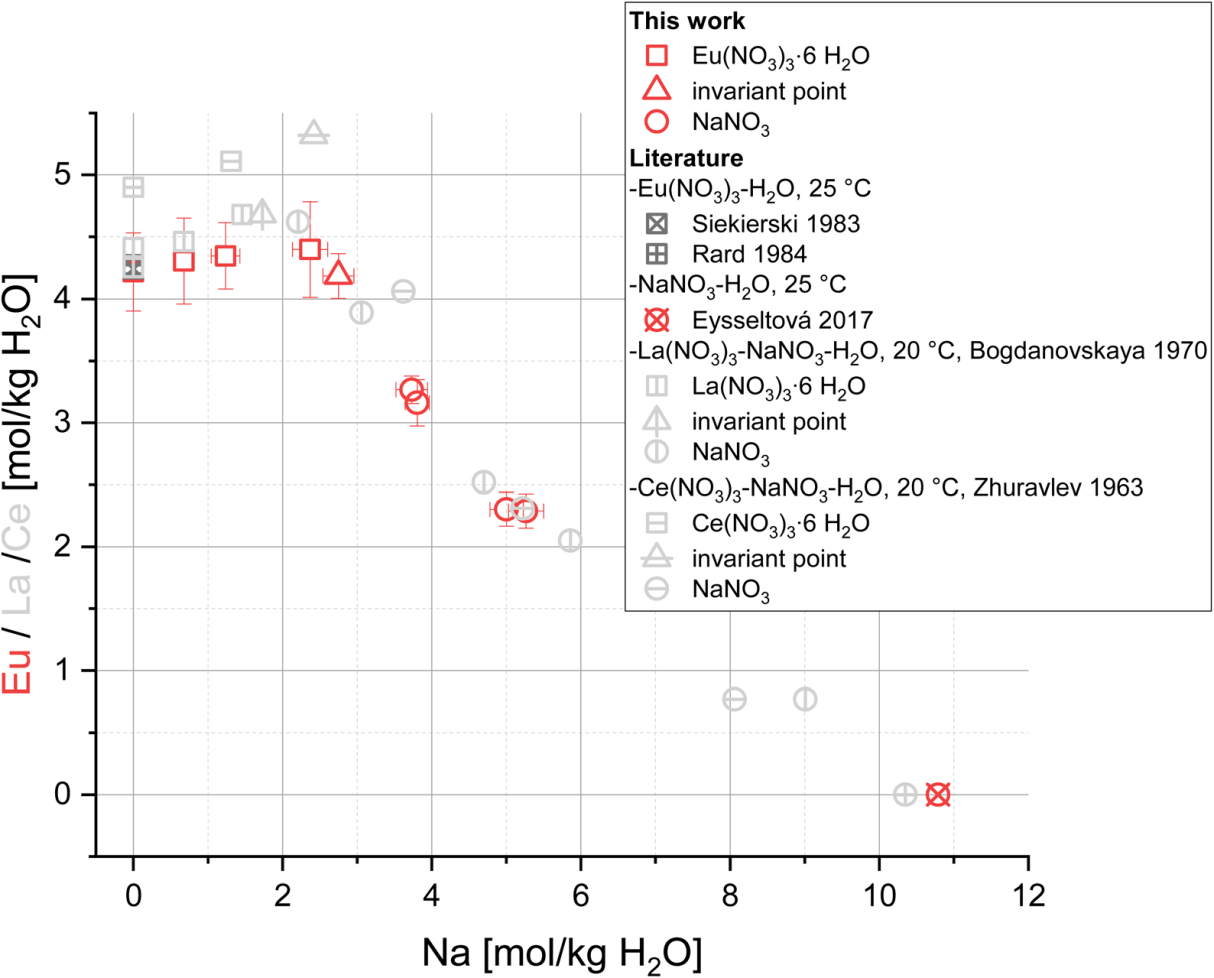
Solubility data for the system Eu(NO_3_)_3_-NaNO_3_-H_2_O (red) at *T* = (22 ± 2) °C (complemented by literature data for solubilities in the binary subsystems at 25 °C ^12–14^) compared to solubility data of the systems La(NO_3_)_3_-NaNO_3_-H_2_O and Ce(NO_3_)_3_-NaNO_3_-H_2_O (light grey).^53,54^

The solubility of Eu(NO_3_)_3_·6H_2_O in water (4.22 ± 0.34 mol per kg H_2_O) is in excellent agreement with literature values.^12,13^ With this concentration baseline, the system Eu(NO_3_)_3_-NaNO_3_-H_2_O (Fig. 1, red symbols) shows a minor increase in Eu concentration up to 4.40 mol per kg H_2_O with increasing NaNO_3_ concentration. The solubility curve reaches an invariant point around 2.75 mol NaNO_3_ per kg H_2_O confirmed *via* XRPD by the simultaneous identification of both solid phases Eu(NO_3_)_3_·6H_2_O and NaNO_3_ in equilibrium with the solution (see Fig. 3 sample D in the next section). Starting from a pure Na-NO_3_-H_2_O system with NaNO_3_ in equilibrium (NaNO_3_ saturation at 10.79 mol per kg H_2_O^14^), NaNO_3_ remains the solid equilibrium phase with increasing the Eu(NO_3_)_3_ concentration until the invariant point (4.18 ± 0.18 mol Eu(NO_3_)_3_ per kg H_2_O) is reached.

The observed trend of the Eu(NO_3_)_3_-NaNO_3_-H_2_O solubility curve is in good agreement with solubility data of the lanthanide systems La(NO_3_)_3_-NaNO_3_-H_2_O^53^ and Ce(NO_3_)_3_-NaNO_3_-H_2_O^54^ (Fig. 1, light grey symbols). Both show a similar increase in lanthanide (La or Ce) concentrations up to their respective invariant points between 1.73 and 2.42 mol NaNO_3_ per kg H_2_O, followed by a continuous decrease in lanthanide concentration with increasing NaNO_3_ concentrations as observed for the Eu(NO_3_)_3_-NaNO_3_-H_2_O system in this work.

**Fig. 2:**
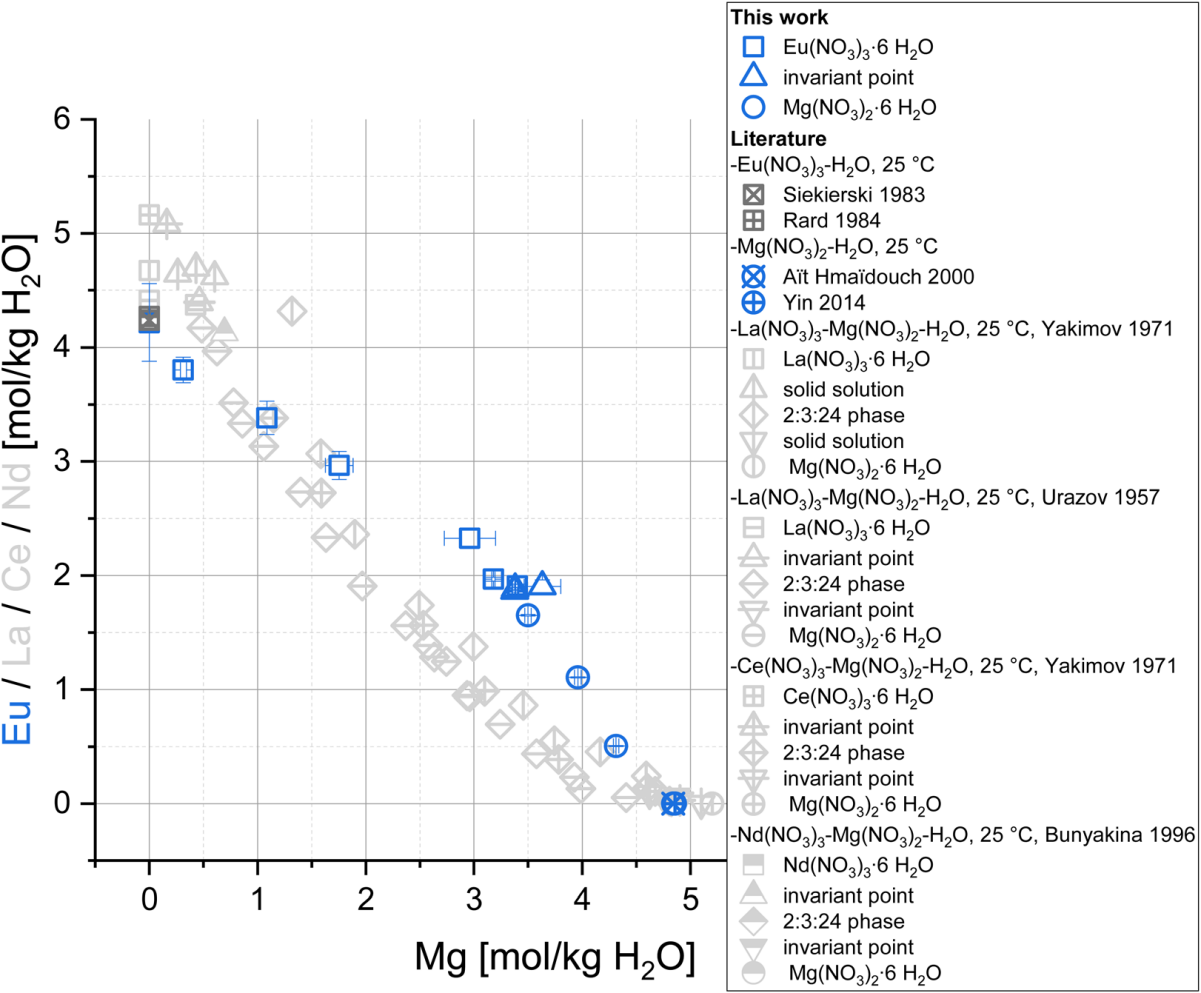
Solubility data for the system Eu(NO_3_)_3_-Mg(NO_3_)_2_-H_2_O (blue) at *T* = (22 ± 2) °C (complemented by literature data for solubilities in the binary subsystems at 25 °C ^12,13,15,16^) compared to solubility data of the systems La(NO_3_)_3_-Mg(NO_3_)_2_-H_2_O, Ce(NO_3_)_3_-Mg(NO_3_)_2_-H_2_O, and Nd(NO_3_)_3_-Mg(NO_3_)_2_-H_2_O (light grey).^53,55,56^

In the case of the Eu(NO_3_)_3_-Mg(NO_3_)_2_-H_2_O system (Fig. 2, blue symbols), Eu concentrations, starting at 4.22 mol per kg H_2_O in the binary Eu(NO_3_)_3_-H_2_O system, decrease continuously with increasing Mg(NO_3_)_2_ concentration up to 4.84–4.86 mol Mg(NO_3_)_2_ per kg H_2_O^15,16^ (saturation concentration of Mg(NO_3_)_2_ in its binary aqueous system). The course of the solubility curve indicates an invariant point of Eu(NO_3_)_3_·6H_2_O and Mg(NO_3_)_2_·6H_2_O at *ca.* 3.4 mol per kg H_2_O Mg(NO_3_)_2_, which overall agrees well with the solid phase analyses by XPRD and Schreinemakers' method (see next section). The formation of 2Eu(NO_3_)_3_·3Mg(NO_3_)_2_·24H_2_O is not evident based on the solubility investigation. This double salt was found in the XRPD pattern of sample K, but cannot be confirmed with Schreinemakers' method. A discussion on this disagreement is provided in the next chapter.

The observed solubility behaviour is significantly different compared to the systems La(NO_3_)_3_-Mg(NO_3_)_2_-H_2_O,^53^ Ce(NO_3_)_3_-Mg(NO_3_)_2_-H_2_O,^55^ and Nd(NO_3_)_3_-Mg(NO_3_)_2_-H_2_O^56^ in which the formation of 2Ln(NO_3_)_3_·3Mg(NO_3_)_2_·24H_2_O (Ln = La, Ce, Nd) dominates between 0.5 and 4.5 mol Mg(NO_3_)_2_ per kg H_2_O (Fig. 2, light grey symbols). Akimov *et al.*^57^ described the formation of the equivalent Eu phase at room temperature from an Eu(NO_3_)_3_-Mg(NO_3_)_2_ solution (concentrations not specified) in 6 molar nitric acid and determined its crystal structure. Qiao *et al.*^58^ investigated phase equilibria in the comparatively acidic (6.2–7.8 mol HNO_3_ per kg H_2_O) quaternary Ln(NO_3_)_3_-Mg(NO_3_)_2_-HNO_3_ (20 w%)-H_2_O systems (Ln = Nd, Sm, Eu, Tb) and reported the formation of 2Ln(NO_3_)_3_·3Mg(NO_3_)_2_·24H_2_O in case of Ln = Nd and Sm but not for Ln = Eu and Tb, based on results of Schreinemakers' method. A significant difference compared to the present work are the high HNO_3_ concentrations in both literature experiments.^57,58^ Comparable solubility studies for the ternary system Eu(NO_3_)_3_-Mg(NO_3_)_2_-H_2_O are not available in the literature, to the best of our knowledge. Based on the overall results, 2Eu(NO_3_)_3_·3Mg(NO_3_)_2_·24H_2_O is assumed to be a metastable phase compared to the single salts in the Eu(NO_3_)_3_-Mg(NO_3_)_2_-H_2_O system with its solubility curve closely passing by the invariant point of Eu(NO_3_)_3_·6H_2_O and Mg(NO_3_)_2_·6H_2_O. In fact, Eu seems to be a “transition point” within the Ln(NO_3_)_3_-Mg(NO_3_)_2_-H_2_O system series, for which the 2Ln(NO_3_)_3_·3Mg(NO_3_)_2_·24H_2_O phase is described as a stable phase over large concentration ranges for lanthanides lighter than Eu, while it is not described as such for heavier ones.

The pH values of all solutions were determined after the last sampling and resulted consequently in values of pH ≤ 3.8. The hydrolysis of Eu(iii) is not expected to play a major role under these conditions as shown by Jordan *et al.*^59^ for the aqueous Eu(iii) hydroxide system. Consequently, Eu^3+^ and Eu(iii)-nitrato complexes are expected as dominant aqueous Eu(iii) species.

Another set of samples was prepared to determine osmotic coefficients at set water activities. Table 2 contains molalities of the involved salts in the ternary systems Eu(NO_3_)_3_-NaNO_3_-H_2_O and Eu(NO_3_)_3_-Mg(NO_3_)_2_-H_2_O. The values were determined by measuring the weight differences for each sample (different initial salt molalities), resulting from equilibration at selected relative humidities RH_eq_
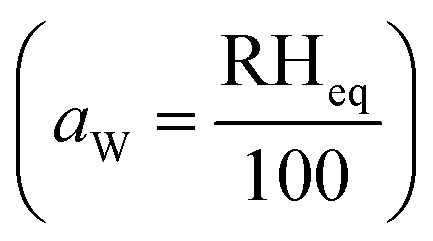
. The osmotic coefficients *φ* were calculated according to eqn (7). The osmotic coefficient values increase with decreasing relative humidity and show reproducibility confirming equilibrium state.

Molalities *m* at set water activities *a*_w_, and resulting osmotic coefficients *φ* in the systems Eu(NO_3_)_3_-NaNO_3_-H_2_O and Eu(NO_3_)_3_-Mg(NO_3_)_2_-H_2_O at 25 °C. IWA: data obtained with the climatic chamber; DVS: data obtained with the DVS apparatus; experimental uncertainty for concentrations below ± 0.05 mol per kg H_2_O

**Table:** 

Eu(NO_3_)_3_-NaNO_3_-H_2_O				
Sample	*a*_w_	m(NaNO_3_) in mol per kg H_2_O	m(Eu(NO_3_)_3_) in mol per kg H_2_O	*φ*
IWA-1	0.60	1.33	3.91	1.550
	0.70	1.07	3.14	1.345
	0.80	0.79	2.32	1.141
	0.60	1.32	3.89	1.557
IWA-2	0.70	2.70	2.72	1.217
	0.80	1.92	1.93	1.073
	0.60	3.45	3.47	1.365
IWA-3	0.80	4.05	1.36	0.914
	0.70	5.80	1.95	1.021
	0.68	6.04	2.03	1.060
DVS-1	0.80	0.72	2.13	1.245
	0.70	0.97	2.86	1.479
	0.60	1.26	3.70	1.637
	0.50	1.52	4.46	1.844
	0.60	1.26	3.71	1.632
	0.70	0.98	2.87	1.475
	0.80	0.74	2.17	1.222
DVS-2	0.80	1.93	1.94	1.064
	0.70	2.59	2.60	1.272
	0.60	3.44	3.46	1.367
	0.50	4.26	4.28	1.501
	0.60	3.48	3.50	1.352
	0.70	2.62	2.63	1.256
DVS-3	0.80	4.00	1.34	0.928
	0.70	5.55	1.86	1.067
	0.60	7.40	2.48	1.146
	0.70	5.71	1.92	1.037
	0.80	4.10	1.37	0.905

**Table:** 

Eu(NO_3_)_3_-Mg(NO_3_)_2_-H_2_O				
Sample	*a*_w_	m(Mg(NO_3_)_2_) in mol per kg H_2_O	m(Eu(NO_3_)_3_) in mol per kg H_2_O	*φ*
DVS-4	0.80	0.60	1.86	1.340
	0.70	0.79	2.45	1.627
	0.60	1.02	3.18	1.795
	0.50	1.24	3.83	2.021
	0.60	1.03	3.20	1.781
	0.70	0.80	2.47	1.612
	0.80	0.60	1.87	1.336
DVS-5	0.80	1.48	1.10	1.399
	0.70	1.95	1.45	1.702
	0.60	2.50	1.86	1.898
	0.50	2.99	2.22	2.158
	0.60	2.51	1.86	1.891
	0.70	1.96	1.45	1.691
	0.80	1.50	1.11	1.386
DVS-6	0.80	1.98	0.65	1.446
	0.70	2.58	0.85	1.777
	0.60	3.29	1.08	1.995
	0.50	3.90	1.29	2.281
	0.60	3.30	1.09	1.990
	0.70	2.59	0.85	1.772
	0.80	1.99	0.66	1.441

### Solid phase characterization

**Fig. 3:**
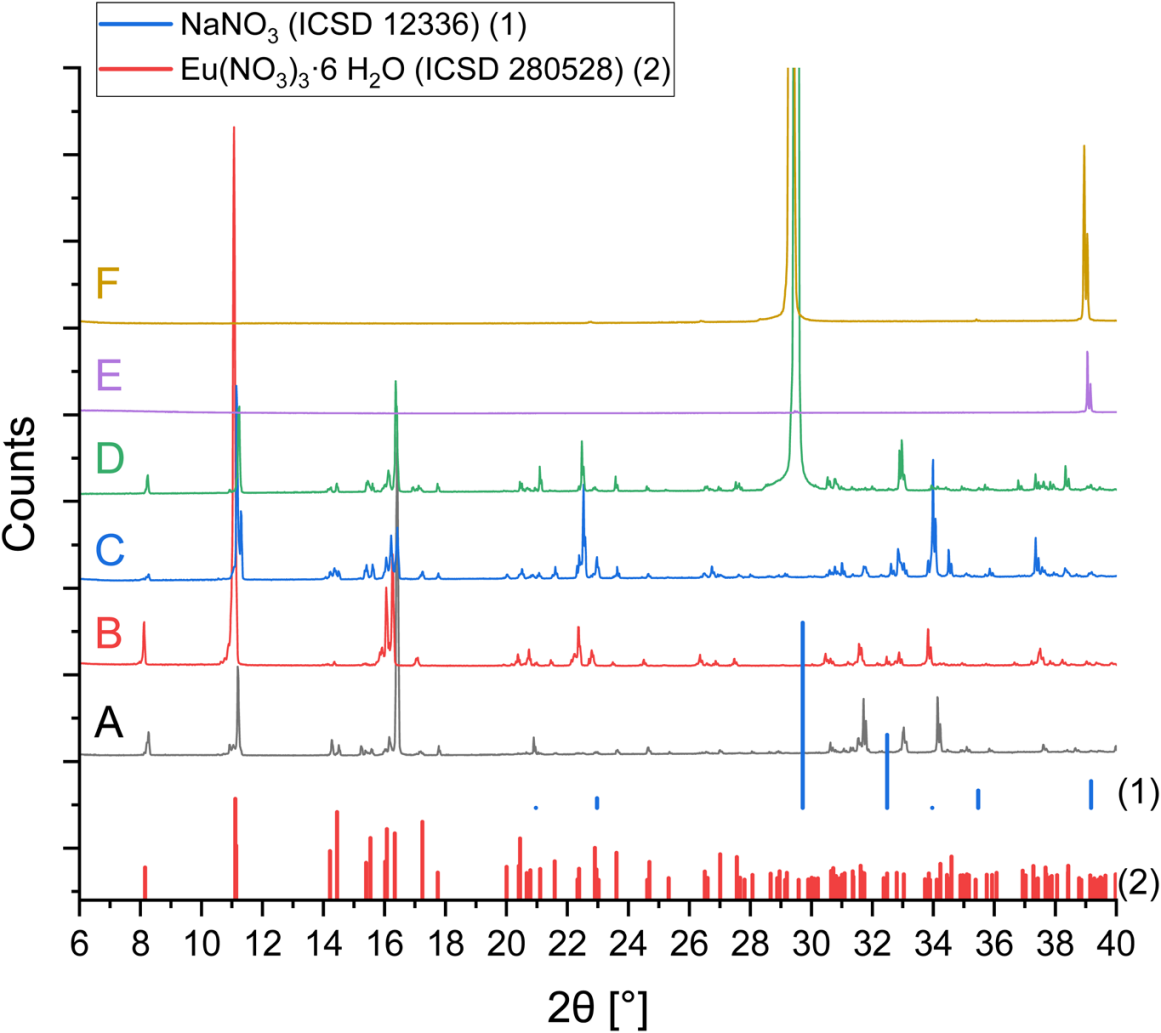
X-ray powder diffraction patterns of the separated solid phases A–F from solubility investigations in the system Eu(NO_3_)_3_-NaNO_3_-H_2_O (0–5 mol NaNO_3_ per kg H_2_O) compared to reference lines.^23,60^

X-ray powder diffraction was used for solid phase characterization of samples from the solubility experiments. The investigated solid samples A–R are indicated in Table 1. Fig. 3 shows the XRPD patterns for samples A–F of the system Eu(NO_3_)_3_-NaNO_3_-H_2_O. Sample A does not contain any NaNO_3_, thus the only solid phase in equilibrium is Eu(NO_3_)_3_·6H_2_O. With rising NaNO_3_ concentration in samples B and C, Eu(NO_3_)_3_·6H_2_O still remains the solid phase in equilibrium. The diffraction pattern of sample D (m(NaNO_3_) = 2.75 mol per kg H_2_O) contains signals of both Eu(NO_3_)_3_·6H_2_O and NaNO_3_, which is consistent with the observed invariant point in the solubility curve. A further increase in NaNO_3_ solution concentrations up to 5 mol per kg H_2_O leads to the sole formation of NaNO_3_, which is the solid phase in equilibrium in samples E and F. These XRPD-results complement the progression of the solubility data in Fig. 1 well.

**Fig. 4:**
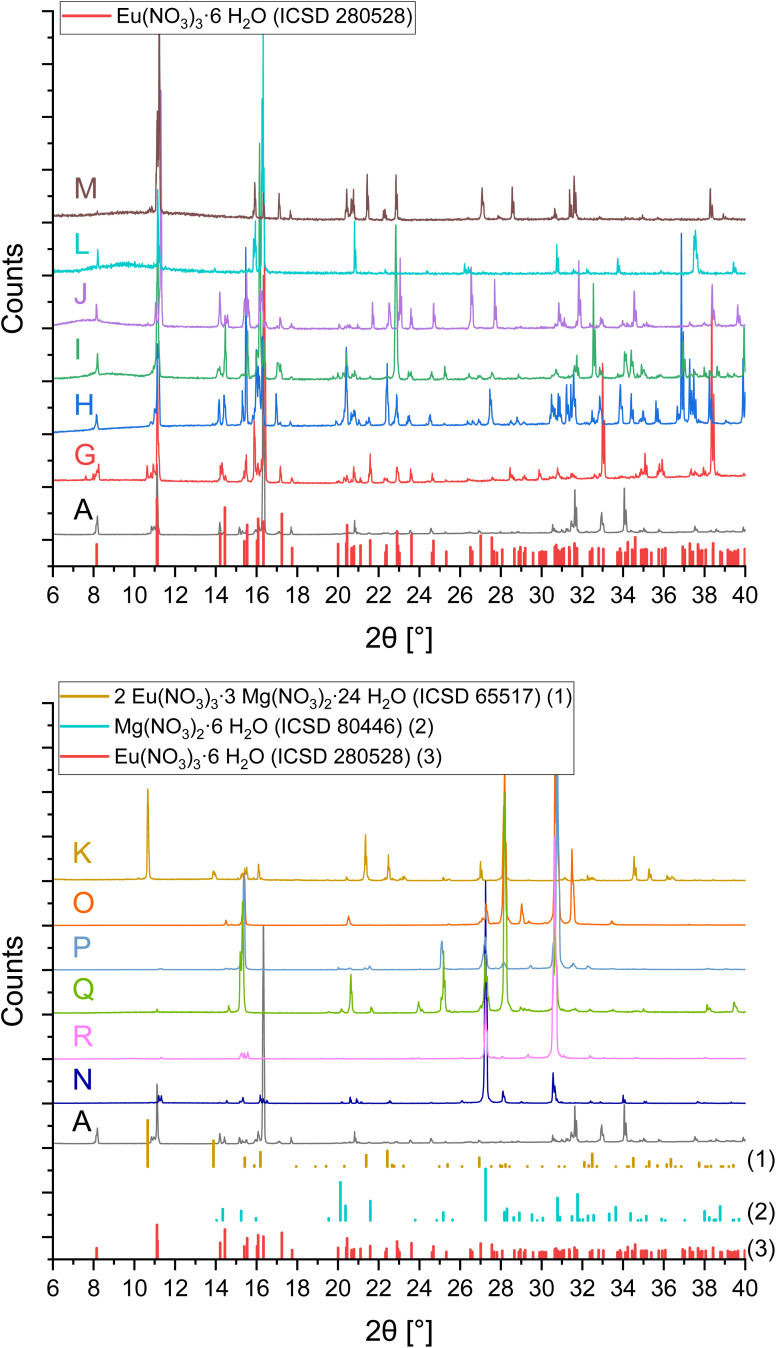
X-ray powder diffraction patterns of the separated solid phases A and G–R from solubility investigations in the system Eu(NO_3_)_3_-Mg(NO_3_)_2_-H_2_O (0–4.3 mol Mg(NO_3_)_2_ per kg H_2_O) compared to reference lines;^23,57,61^ top: Eu(NO_3_)_3_·6H_2_O dominated samples (≤3.4 mol Mg(NO_3_)_2_ per kg H_2_O in solution), bottom: Mg(NO_3_)_2_·6H_2_O dominated samples (≥3.4 mol Mg(NO_3_)_2_ per kg H_2_O in solution); slight 2*θ*-shifts are executed for better illustration and comparison among the patterns (unmodified and time dependent XRPD results are included in the SI).

The XRPD patterns for samples of the system Eu(NO_3_)_3_-Mg(NO_3_)_2_-H_2_O are illustrated in Fig. 4 in comparison to sample A. The patterns in the upper part (samples G–J, L, M) can be described with the Eu(NO_3_)_3_·6H_2_O reference lines.^23^ The XRPD patterns in the lower part of Fig. 4 (samples K and O–R) are distinctly different, which indicates a solid phase change in solutions with ≥3.4 mol Mg(NO_3_)_2_ per kg H_2_O (stated invariant point based on the determined solubility curve).

The diffraction patterns of samples N–R are best explained by Mg(NO_3_)_2_·6H_2_O reference lines.^61^ The presence of Eu(NO_3_)_3_·6H_2_O is expected for samples N and R based on the composition of their corresponding solutions, but is in the XRPD patterns just indicated by minor signals. Solution compositions of samples O, P and Q are on the assumed Mg(NO_3_)_2_·6H_2_O-branch of the solubility curve, where Mg(NO_3_)_2_·6H_2_O should be the solid phase in equilibrium, which is consistent with the X-ray diffraction patterns.

The XRPD pattern of sample K (10.7 °2*θ*, 21.4 °2*θ*, and 22.4 °2*θ*, …) can best be explained by 2Eu(NO_3_)_3_·3Mg(NO_3_)_2_·24H_2_O reference lines.^57^ Based on the solubility experiments, this is expected to be a metastable phase compared to the simple Eu- and Mg-nitrate salts, since the corresponding solution of this sample is supersaturated compared to the systematic solubility curve which results from the solution compositions of all other samples. This indicates that the formation is also the result of a, at least local, oversaturation. Note however that a phase transformation of this once formed 2:3:24 phase in sample K was not observed in the course of these experiments (up to 2 years). This might be due to kinetic inhibition but is not clear for the time being, and should be further investigated in the future. The presence of Mg(NO_3_)_2_·6H_2_O cannot be excluded based on the XRPD results, due to the overlap of some reference lines.

XRPD measurements overall provided some experimental challenges. Different attempts of washing the solid phases (with water or ethanol) resulted in phase changes and dissolution due to high solubility of nitrates. Strong hygroscopic effects led to dissolution of initially dried samples during longer measurements. Thus, comparatively short measurements of roughly crushed crystals with adherent mother liquor were executed to qualitatively analyse the solids, before any phase change. This results in signal displacements and missing signals due to preferential crystal orientations (Fig. 4). However, the results still provide valuable information on the solid phases.

Rietveld refinements were conducted for samples A–J. Resulting compositions are listed in Table 1, while refined unit cell parameters, sizes of coherent scattering domains, and all Rietveld plots can be found in the SI. For samples with the equilibrium phase Eu(NO_3_)_3_·6H_2_O (A–D, G–J) it was shown that despite Eu(NO_3_)_3_·6H_2_O being the main phase, Eu(NO_3_)_3_(H_2_O)_4_(H_2_O) was always found in varying amounts. This indicates a secondary phase change during sample preparation and XRD measurement. Both phases are quite similar and show strong orientation. NaNO_3_ was found in samples D–F, identified with *ca.* 17 wt% in sample D (invariant point of the solubility curve; the theoretical ratio of NaNO_3_ in a 1 : 1 mixture with Eu(NO_3_)_3_·6H_2_O is 16 wt%), while being the only solid in equilibrium for samples E and F.

**Fig. 5:**
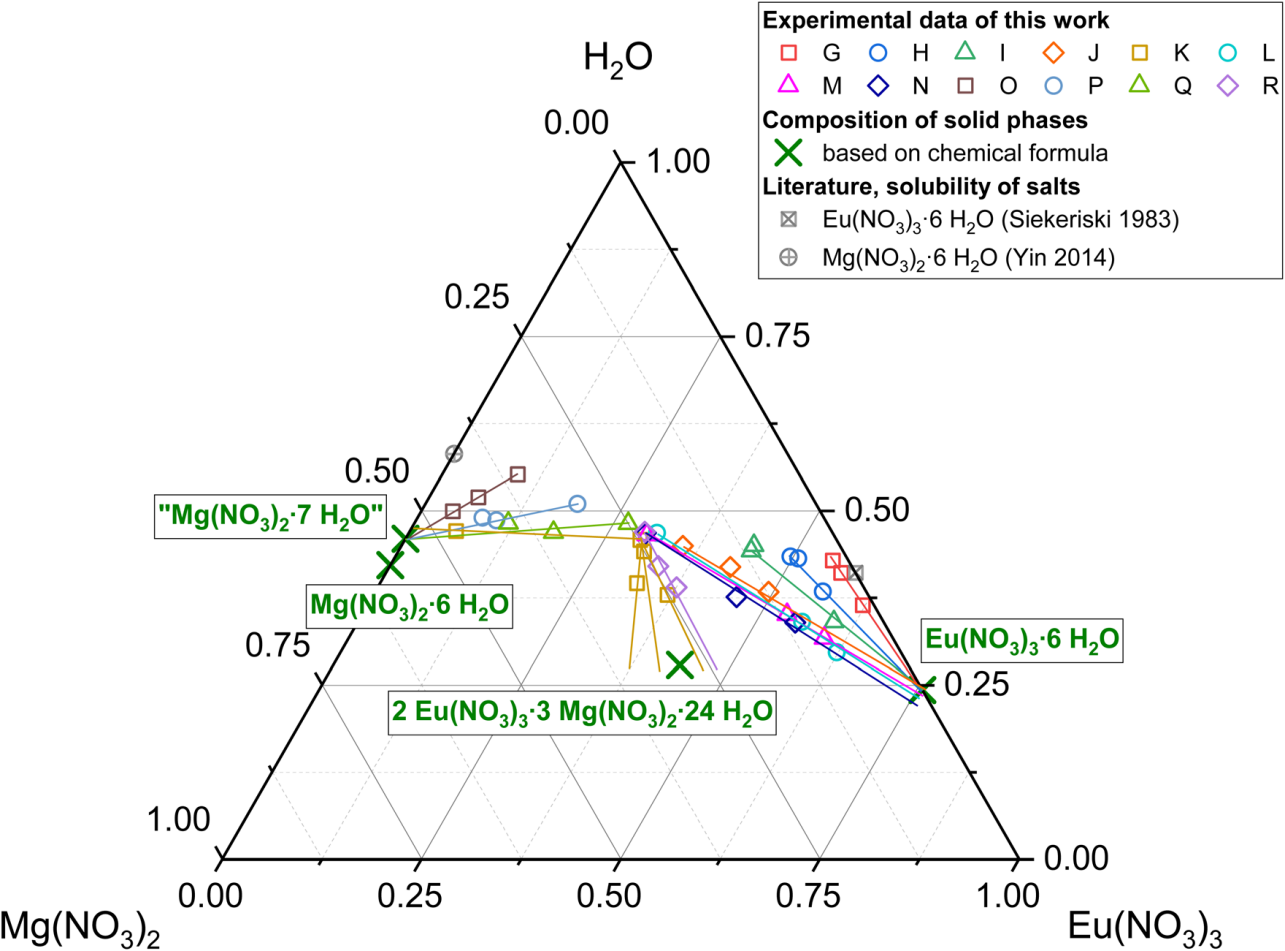
Schreinemakers' method to determine the composition of solid phases in equilibrium with the solutions of samples G–R in the system Eu(NO_3_)_3_-Mg(NO_3_)_2_-H_2_O (see Table 3); data points of the same sample are illustrated in the same colour and include compositions of separated solutions and suspensions with different solid/liquid ratios, lines are added manually for illustration; grey symbols represent literature solubility data^12,16^ of Mg(NO_3_)_2_·6H_2_O and Eu(NO_3_)_3_·6H_2_O at 25 °C in their respective binary aqueous systems.

Because of the experimental issues for XRPD and the uncertainties coming with them for the Eu(NO_3_)_3_-Mg(NO_3_)_2_-H_2_O system, solid phase compositions were additionally analysed by Schreinemakers' method,^27^ in which the composition of solid phases is graphically deduced based on the composition of the solution in equilibrium and corresponding suspensions. At least three points were determined per sample to provide more analytical accuracy despite the high dilutions necessary for analysis. Results are listed in Table 3 and illustrated in Fig. 5.

**Table 3:** Composition (in mass fraction) of samples for determination of solid phases in the system Eu(NO_3_)_3_-Mg(NO_3_)_2_-H_2_O at *T* = (22 ± 2) °C using Schreinemakers' method; corresponding mass fractions of water were calculated based on these values

Sample	Solution		Suspension 1 (low solid/liquid ratio)		Suspension 2 (high solid/liquid ratio)	
	Eu(NO_3_)_3_	Mg(NO_3_)_2_	Eu(NO_3_)_3_	Mg(NO_3_)_2_	Eu(NO_3_)_3_	Mg(NO_3_)_2_
G	0.551 ± 0.005	0.020 ± 0.002	0.571 ± 0.011	0.018 ± 0.001	0.621 ± 0.009	0.014 ± 0.001
H	0.496 ± 0.011	0.070 ± 0.005	0.507 ± 0.008	0.062 ± 0.002	0.562 ± 0.008	0.055 ± 0.001
I	0.443 ± 0.007	0.115 ± 0.008	0.443 ± 0.003	0.108 ± 0.002	0.598 ± 0.010	0.062 ± 0.001
J	0.354 ± 0.009	0.197 ± 0.016	0.428 ± 0.015	0.15 ± 0.0060	0.494 ± 0.006	0.123 ± 0.003
K	0.295 ± 0.009	0.247 ± 0.012	0.308 ± 0.004	0.250 ± 0.006	0.322 ± 0.003	0.282 ± 0.004
			0.369 ± 0.002	0.252 ± 0.002	0.058 ± 0.001	0.472 ± 0.005
L	0.311 ± 0.003	0.221 ± 0.001	0.558 ± 0.009	0.102 ± 0.001	0.623 ± 0.002	0.081 ± 0.001
M	0.300 ± 0.003	0.235 ± 0.001	0.534 ± 0.003	0.116 ± 0.001	0.597 ± 0.003	0.087 ± 0.001
N	0.298 ± 0.002	0.235 ± 0.002	0.458 ± 0.002	0.167 ± 0.002	0.550 ± 0.002	0.112 ± 0.001
O	0.094 ± 0.001	0.353 ± 0.002	0.061 ± 0.001	0.419 ± 0.003	0.039 ± 0.001	0.461 ± 0.002
P	0.191 ± 0.001	0.300 ± 0.002	0.101 ± 0.001	0.413 ± 0.004	0.081 ± 0.002	0.429 ± 0.004
Q	0.269 ± 0.001	0.250 ± 0.002	0.181 ± 0.001	0.350 ± 0.006	0.118 ± 0.001	0.400 ± 0.001
R	0.296 ± 0.002	0.235 ± 0.002	0.337 ± 0.003	0.243 ± 0.002	0.375 ± 0.003	0.235 ± 0.004

Eu(NO_3_)_3_·6H_2_O can be identified as solid phase in equilibrium for samples G–J, L, and M, which agrees well with the results of XRPD (Fig. 4 top). Furthermore, it becomes evident that the solid phase in equilibrium with samples O, P and Q is a magnesium nitrate hydrate. Considering the present analytical uncertainty and the XRPD results, it is reasonable to assign Mg(NO_3_)_2_·6H_2_O rather than Mg(NO_3_)_2_·7H_2_O, despite deviation from its exact hydration water content.

The results of the remaining samples N, R and K do not seem to describe a single solid phase, but a mixture of solids, which agrees with their solution compositions close to the postulated invariant point of the solubility curve. For samples N and R, a mixture of Mg(NO_3_)_2_·6H_2_O and Eu(NO_3_)_3_·6H_2_O is deduced based on the location of their data points comparatively close to Eu(NO_3_)_3_·6H_2_O in combination with the identification of Mg(NO_3_)_2_·6H_2_O in the XRPD patterns. The presence of 2Eu(NO_3_)_3_·3Mg(NO_3_)_2_·24H_2_O was already shown for sample K by XRPD investigations. The scattering of its data points in Schreinemakers' method is based on varying ratios of different solids in the different samplings, which is most likely Mg(NO_3_)_2_·6H_2_O besides 2Eu(NO_3_)_3_·3Mg(NO_3_)_2_·24H_2_O.

### Thermodynamic modelling

The parameters required for the full dissociation models of the ternary systems Eu(NO_3_)_3_-NaNO_3_-H_2_O and Eu(NO_3_)_3_-Mg(NO_3_)_2_-H_2_O are summarized in Table 4. Pitzer parameters for the binary systems Eu(NO_3_)_3_-H_2_O, Mg(NO_3_)_2_-H_2_O, and NaNO_3_-H_2_O as well as corresponding solubility constants 
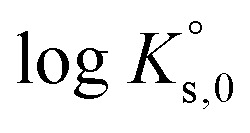
 are already established in the PhreeSCALE database.^32^ They are taken from the works of Guignot *et al.*^41^ and Lach *et al.*,^42^ which provide comprehensive reviews on existing data as well as model attempts and present parameter sets most suitable for describing mean activity coefficients and osmotic coefficients at all concentrations up to saturation.

Parameters used for modelling the systems Eu(NO_3_)_3_-NaNO_3_-H_2_O and Eu(NO_3_)_3_-Mg(NO_3_)_2_-H_2_O at 25 °C in this work

**Table:** 

Pitzer parameters binary systems							
Species *i*	Species *j*	*α*_1/2_	*β* ^(0)^_*i*,*j*_	*β* ^(1)^_*i*,*j*_	*β* ^(2)^_*i*,*j*_	*C*_*i*,*j*_^*ϕ*^	References
Eu^3+^	NO_3_^−^	2/0.6424	0.2669	3.5169	0.7029	−0.0072	Guignot *et al.*^41^
Na^+^	NO_3_^−^	—	0.0038	0.1835	—	0	Lach *et al.*^42^
Mg^2+^	NO_3_^−^	—	0.3286	1.9159	—	−0.0064	Lach *et al.*^42^

**Table:** 

Pitzer parameters ternary systems					
Species *i*	Species *j*	Species *k*	*θ*_*i*,*k*_	*Ψ*_*i*,*k*,*j*_	References
Eu^3+^	NO_3_^−^	Na^+^	0.2223*	−0.017**	*F. dos Santos *et al.*^34^
					**This work
Eu^3+^	NO_3_^−^	Mg^2+^	−0.182*	0.017**	*F. dos Santos *et al.*^34^
					**This work

**Table:** 

Solubility constants		
Solid phase	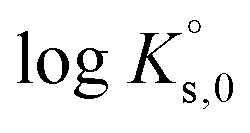	References
Eu(NO_3_)_3_ 6H_2_O ⇄ Eu^3+^+3NO_3_^−^ + 6H_2_O	1.8617	Guignot *et al.*^41^
NaNO_3_ ⇄ Na^+^ + NO_3_^−^	1.0864	Lach *et al.*^42^
Mg(NO_3_)_2_·6H_2_O ⇄ Mg^2+^ + 2NO_3_^−^ + 6H_2_O	3.05	Lach *et al.*^42^

In order to describe the ternary systems properly, further Pitzer parameters *θ*_*i*,*k*_ and *Ψ*_*i*,*k*,*j*_ need to be added. These describe interactions of the different cations *i* and *k* (Eu^3+^ and Na^+^ or Mg^2+^) as well as the ternary interactions between cations *i* and *k* with the anion *j* (NO_3_^−^). The parameters *θ*_*i*,*k*_ were taken from the recent work of F. dos Santos *et al.*,^34^ which provides full dissociation Pitzer models for the equivalent sulphate systems. The parameter sets were completed by determining the *Ψ*_*i*,*k*,*j*_ parameters in this work, based on the presented experimental data.

**Fig. 6:**
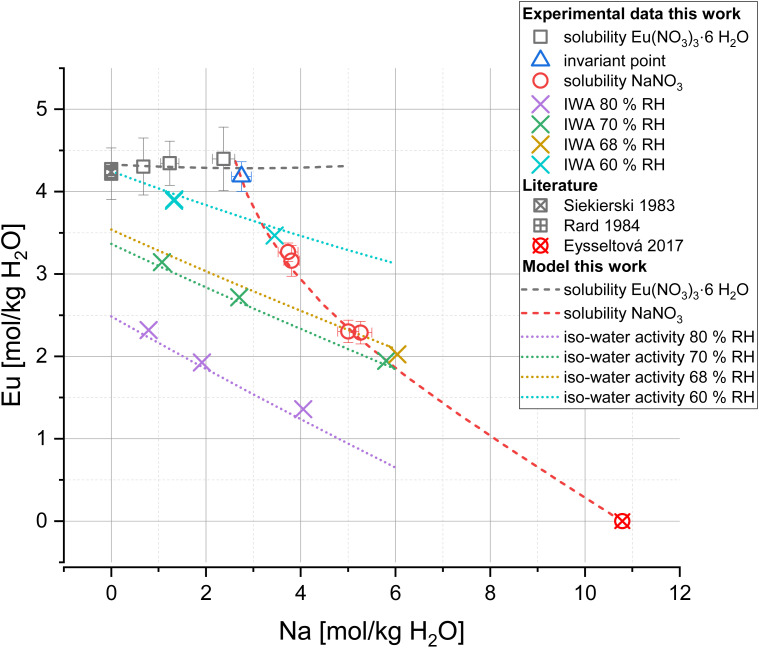
Eu(NO_3_)_3_-NaNO_3_-H_2_O solubility and iso-water activity curves at 25 °C calculated with PhreeSCALE (lines) compared to experimental data of this work and literature^12–14^ (symbols).

Fig. 6 shows the solubility and iso-water activity data (symbols) compared to the model calculations (lines) for the system Eu(NO_3_)_3_-NaNO_3_-H_2_O. The modelled solubility curves reproduce the experimental values within the experimental uncertainties including the location of the invariant point Eu(NO_3_)_3_-NaNO_3_ at 2.75 mol NaNO_3_ per kg H_2_O. The calculated iso-water activity curves are also in good agreement with the experimental results, illustrating the linear correlation at each relative humidity. The respective sigma values of 0.071 for solubility data and 0.084 for osmotic coefficients further indicate an overall good match between model and experimental results.

**Fig. 7:**
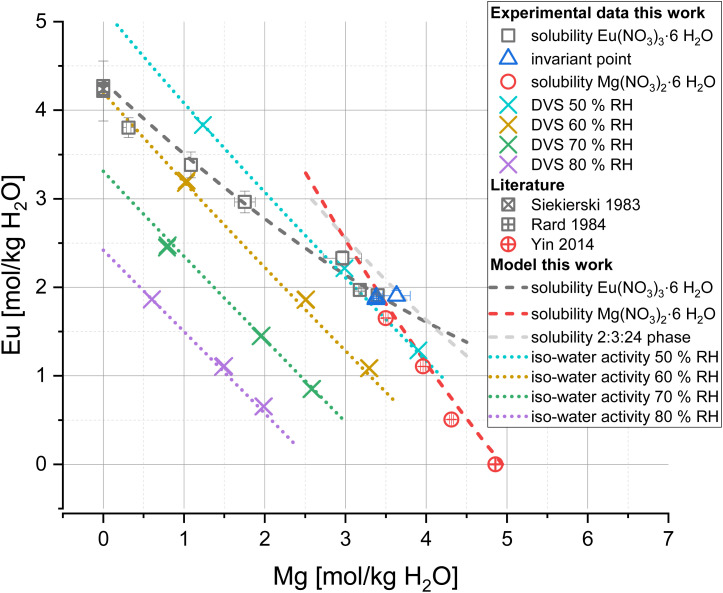
Eu(NO_3_)_3_-Mg(NO_3_)_2_-H_2_O solubility and iso-water activity curves at 25 °C calculated with PhreeSCALE (lines) compared to experimental data of this work and literature^12,13,15,16^ (symbols).

The comparison of experiments and the full dissociation Pitzer model for the system Eu(NO_3_)_3_-Mg(NO_3_)_2_-H_2_O is shown in Fig. 7. An overall good agreement between experimental and calculated results is given for iso-water activity and solubility data (sigma values of 0.053 and 0.141, respectively). The linear trend of the iso-water activity data was reproduced for all investigated relative humidities. The solubility data of Eu(NO_3_)_3_·6H_2_O and Mg(NO_3_)_2_·6H_2_O are also properly reproduced including their invariant point around 3.4 mol Mg(NO_3_)_2_ per kg H_2_O. Based on the presence of metastable 2Eu(NO_3_)_3_·3Mg(NO_3_)_2_·24H_2_O in sample K, an additional solubility curve for this ternary 2:3:24 phase (Fig. 7, grey line) was calculated based on an estimated solubility product, *i.e.*, 
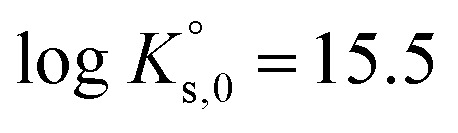
. Note that this value does not represent equilibrium conditions and should not be further implemented in thermodynamic databases.

Zalupski *et al.*^44^ presented a large set of osmotic coefficients at 25 °C for the system Eu(NO_3_)_3_-NaNO_3_-H_2_O (up to 1 mol Eu per kg H_2_O and 1.5 mol Na per kg H_2_O), which is also reproduced by the presented model in very good approximation (*σ*(*φ*) = 0.008; see Fig. SI-20 in the SI). It should be mentioned that they introduced their own set of binary and ternary Pitzer parameters (comparable in magnitude to the binary and ternary parameters in Table 4). Although it describes their osmotic coefficients well, the parameters in Table 4 were preferred within the scope of this work to cover a larger concentration range of Eu and Na, and to keep consistency within the used database.

Lassin *et al.*^62^ determined interaction parameters *θ*_*i*,*k*_ and *Ψ*_*i*,*k*,*j*_ (based on solubility data published in Silcock *et al.*^63^) for related ternary lanthanide-nitrate systems Cm(NO_3_)_3_-NaNO_3_-H_2_O (*θ*_Cm^3+^,Na^+^_ = 0.16623 and *Ψ*_Cm^3+^,Na^+^,NO_3_^−^_ = −0.00936) and La(NO_3_)_3_-Mg(NO_3_)_2_-H_2_O (*θ*_La^3+^,Mg^2+^_ = −0.68470 and *Ψ*_La^3+^,Mg^2+^,NO_3_^−^_ = 0.00501), which are in a comparable magnitude to the parameters of this work for the respective Eu systems.

**Fig. 8:**
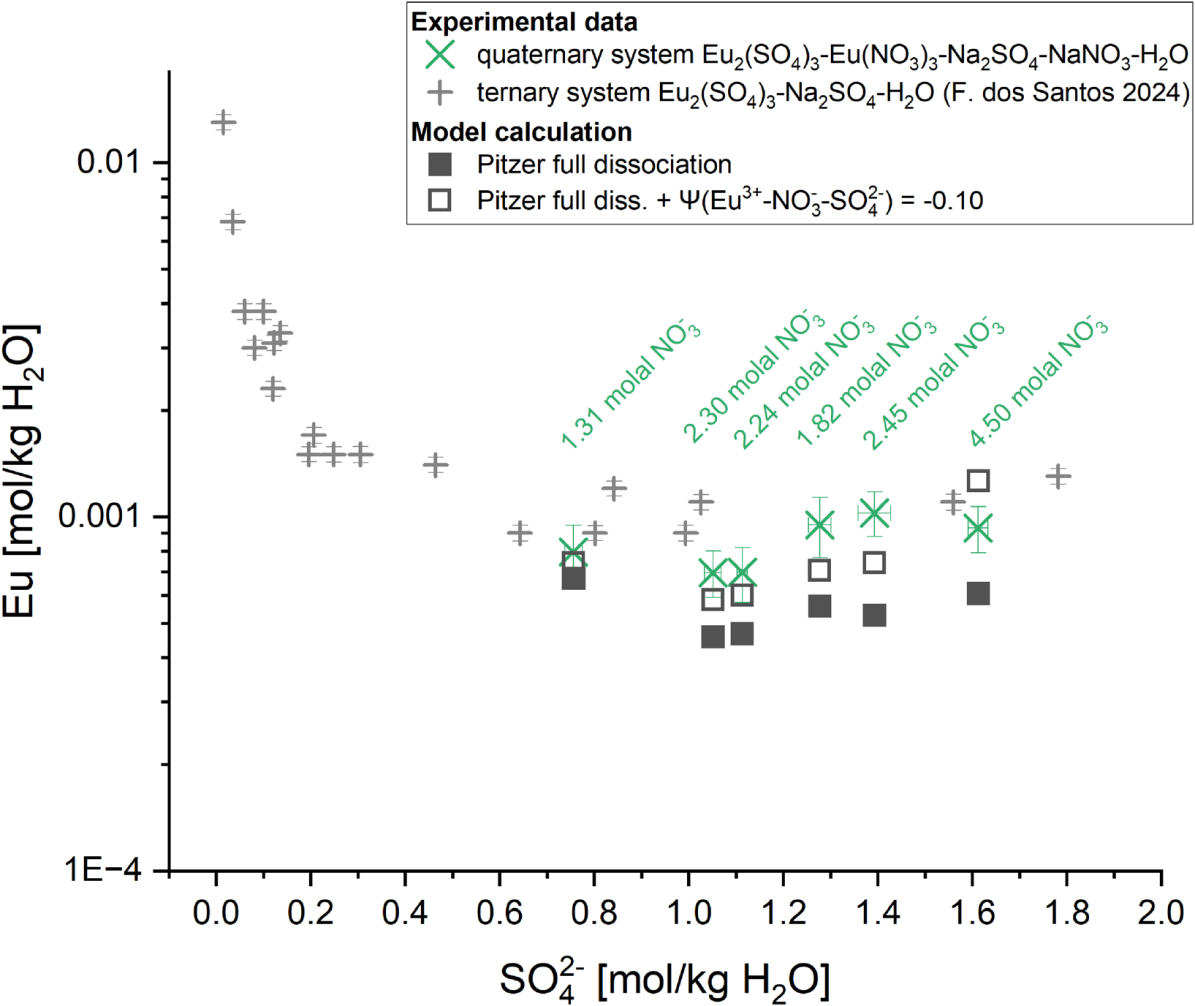
Eu solubility data at 25 °C in the system Eu_2_(SO_4_)_3_-Eu(NO_3_)_3_-Na_2_SO_4_-NaNO_3_-H_2_O calculated with PhreeSCALE (full black squares) compared to experimental data of this work (green crosses; NO_3_^−^ concentrations labelled at each point); open symbols show the same calculations with the introduction of *Ψ*_Eu^3+^,NO_3_^−^,SO_4_^2−^_ = −0.1 to illustrate the impact of this currently unknown parameter on calculations in the quaternary system; grey crosses show experimental solubility data from the ternary system Eu_2_(SO_4_)_3_-Na_2_SO_4_-H_2_O (F. dos Santos *et al.*^34^).

The presented parameter set is proposed as an addition to the PhreeSCALE database for the description of ternary Eu(iii)–nitrate systems. It continues a series started with the respective Eu(iii)–sulphate systems^34,64^ to enable a thermodynamic description of high saline Ln(iii)/An(iii)–sulphate–nitrate systems. Additional solubility experiments were conducted within the quaternary system Eu_2_(SO_4_)_3_-Eu(NO_3_)_3_-Na_2_SO_4_-NaNO_3_-H_2_O to test the progress to this point. The experiments included six samples with varying sulphate and nitrate contents to cover a wide concentration range. The resulting Eu concentrations are plotted against sulphate concentrations in Fig. 8 (results are listed in Table 5). In comparison, calculations using the PhreeSCALE database with the additional parameters of the full dissociation Pitzer models for the ternary systems Eu_2_(SO_4_)_3_-Na_2_SO_4_-H_2_O (F. dos Santos *et al.*^34^) and Eu(NO_3_)_3_-NaNO_3_-H_2_O (this work) are presented (full squares).

**Table 5:** Solubility data for the quaternary system Eu_2_(SO_4_)_3_-Eu(NO_3_)_3_-Na_2_SO_4_-NaNO_3_-H_2_O at *T* = (22 ± 2) °C; NO_3_^−^ values are calculated based on weigh-ins under the assumption of full nitrate solubility and complete formation of NaEu(SO_4_)_2_·H_2_O

Sample	Eu^3+^ in mmol per kg H_2_O	Na^+^ in mol per kg H_2_O	SO_4_^2−^ in mol per kg H_2_O	NO_3_^−^ in mol per kg H_2_O
1	0.80 ± 0.15	2.78 ± 0.06	0.76 ± 0.02	1.31
2	0.70 ± 0.10	4.34 ± 0.05	1.05 ± 0.02	2.30
3	0.70 ± 0.12	4.47 ± 0.07	1.11 ± 0.01	2.24
4	1.03 ± 0.15	5.17 ± 0.14	1.39 ± 0.03	2.45
5	0.93 ± 0.14	7.55 ± 0.07	1.61 ± 0.02	4.50
6	0.95 ± 0.18	4.33 ± 0.03	1.28 ± 0.02	1.82

The calculated solid phase in equilibrium within the investigated sulphate concentration range is the double salt NaEu(SO_4_)_2_·H_2_O, which agrees well with the results of F. dos Santos *et al.*^34^ at the sulphate concentrations investigated in this work. The current model results in slightly lower Eu(iii) concentrations at the given sodium nitrate and sulphate concentrations compared to the experimental data. The fit can be improved by introducing the missing ternary parameter *Ψ*_Eu^3+^,NO_3_^−^,SO_4_^2−^_, which describes the interactions between the cation Eu^3+^ and the anions NO_3_^−^ and SO_4_^2−^ (interfering anion interactions with Eu^3+^ or competitive influence of the anions on Eu^3+^). Introducing this parameter directly influences the Eu concentration in the quaternary system (exemplified for *Ψ*_Eu^3+^,NO_3_^−^,SO_4_^2−^_ = −0.1; open symbols in Fig. 8). The value of *Ψ*_Eu^3+^,NO_3_^−^,SO_4_^2−^_ determined in this work should be considered as tentative due to the limited dataset available (6 experimental points). Future dedicated studies on the ternary system Eu_2_(SO_4_)_3_-Eu(NO_3_)_3_-H_2_O resulting in comprehensive datasets should be the basis for an accurate determination of this parameter.

## Summary and conclusions

Solubility experiments in the ternary systems Eu(NO_3_)_3_-NaNO_3_-H_2_O and Eu(NO_3_)_3_-Mg(NO_3_)_2_-H_2_O were conducted at *T* = (22 ± 2) °C over concentrations ranges of 0.0–5.3 mol NaNO_3_ per kg H_2_O and 0.0–4.3 mol Mg(NO_3_)_2_ per kg H_2_O in weakly acidic solutions. Solid phase characterization was performed by X-ray powder diffraction and Schreinemakers' method. Eu(NO_3_)_3_·6H_2_O and NaNO_3_ were the only solid phases identified over the complete concentration range in the case of Eu(NO_3_)_3_-NaNO_3_-H_2_O. The invariant point observed in the solubility curve at 2.75 mol NaNO_3_ per kg H_2_O was confirmed by XRPD. Eu(NO_3_)_3_·6H_2_O and Mg(NO_3_)_2_·6H_2_O are defined as the only thermodynamically stable phases in the Eu(NO_3_)_3_-Mg(NO_3_)_2_-H_2_O system. Solubility experiments and XRPD confirm the presence of an invariant point at *ca.* 3.4 mol Mg(NO_3_)_2_ per kg H_2_O. The formation of 2Eu(NO_3_)_3_·3Mg(NO_3_)_2_·24H_2_O was observed in one sample with a solution composition close to this invariant point, but this phase is considered as metastable at *T* = (22 ± 2) °C. Additional iso-water activity experiments at *T* = 25 °C were performed for a better characterization of ion interactions in solution by determining osmotic coefficients at fixed relative humidities (50–80% RH) for both ternary systems.

These results enabled the determination of a new set of Pitzer interaction parameters, which accurately describes solubility and iso-water activity data of both investigated systems at *T* = 22–25 °C and within the solubility limits of the corresponding salts. Based on the solubility constants and binary interaction parameters already implemented in PhreeSCALE^32^ we derived missing ternary *Ψ*_*i*,*k*,*j*_ parameters with the help of 70 new experimental data points, consistently with the *θ*_*i*,*k*_ cation–cation interaction parameters determined earlier by F. dos Santos *et al.*^34^ Although out of the scope of this work, the need of extending the current model to elevated temperatures (*T* ≤ 100 °C) with the corresponding set of temperature-dependent Pitzer coefficients is emphasized.

The presented thermodynamic model assumes the full dissociation of salts to Eu^3+^, Mg^2+^, Na^+^ and NO_3_^−^ ions. This drastically reduces the number of parameters, whose values however implicitly need to balance complexation effects expected in solution. Future work will incorporate spectroscopic data to target the description of complex formations in the ternary systems Eu(NO_3_)_3_-NaNO_3_-H_2_O and Eu(NO_3_)_3_-Mg(NO_3_)_2_-H_2_O. A combination of the presented experimental results of this work and time resolved laser fluorescence spectroscopy data enables the derivation of alternative SIT and Pitzer models, which consider the formation of aqueous complexes explicitly.

## Conflicts of interest

There are no conflicts to declare.

## Supplementary Material

RA-016-D5RA09321J-s001

## Data Availability

Additional data supporting this article have been included as part of the supplementary information (SI). Supplementary information: include additional XRD measurements, Rietveld refinement results, and the comparison of the full dissociation model results with osmotic coefficient data from literature. See DOI: https://doi.org/10.1039/d5ra09321j.
