# Supplementary material for: Thermodynamic description of Eu(NO3)3-NaNO3-H2O and Eu(NO3)3-Mg(NO3)2-H2O systems. Solubility experiments and full dissociation Pitzer models
Source: RSC Adv. 2026 May 20;16(28):25927–43. doi: 10.1039/d5ra09321j (PMC13191746; doi:10.1039/d5ra09321j)
Supplement: RA-016-D5RA09321J-s001 [file RA-016-D5RA09321J-s001.pdf]

Supplementary Information (SI) for RSC Advances.
This journal is © The Royal Society of Chemistry 2026

Supporting Information:

Thermodynamic description of the Eu(III)-Na-NO_3-H_2O and Eu(III)-Mg-NO_3-H_2O systems. Part I: solubility experiments and full dissociation Pitzer models

F. Häusler[1,*], P. F. dos Santos[1], A. Lassin[2,*], X. Gaona[1], K. Garbev[3], S. Touzelet[2], Y. Cartigny[4], M. Altmaier[1] and B. Madé[5]

[1]*Institute for Nuclear Waste Disposal, Karlsruhe Institute of Technology, Karlsruhe, Germany*

[2]*BRGM, F-45060 Orléans, France*

[3]*Institute for Technical Chemistry, Karlsruhe Institute of Technology, Karlsruhe, Germany*

[4]*SMS, Univ. Rouen Normandie, F-76821 Mont Saint Aignan, France*

[5]*Scientific and Technical Division, Andra, Châtenay-Malabry, France*

**corresponding author(s): felix.haeusler@kit.edu, a.lassin@brgm.fr*

X-ray powder diffraction measurements

X-ray powder diffraction measurements were challenged by the high solubility and hygroscopy of nitrate salts, as well as the observed phase changes during measurements. To receive qualitative information on the solid phases, sample preparation was kept as short as possible. Washing with ethanol was tested for sample L (Fig. SI-2) and led to dissolution and recrystallization processes resulting in changes in the diffraction powder patterns (formation of $Eu(NO_3)_3 \cdot 5\ H_2O$ besides $Eu(NO_3)_3 \cdot 6\ H_2O$) compared to measuring the samples without prior washing steps (Fig. SI-1). Thus, it was decided to do short measurements without washing directly after separation from the solution to gain qualitative information on the solid phase assemblage. Since most uncertainty remained in the system Eu(III)-Mg-NO_3-H_2O, Samples K-R were measured three times in a row to observe possible changes during the measurement time. All of these measurements, without any adjustments, are shown in Figures SI-1-9. It is necessary to keep in mind that pattern shifts and missing signals due to preferred orientations are a common issue without sample preparation that needs to be considered in the evaluation. Generally, assigned references have to match diffraction patterns and not just individual signals. The XRPD results are under these circumstances indications on the solid phase assemblage and need to be assessed in combination with the solubility investigations and the results of Schreinemakers' method to obtain coherent conclusions.

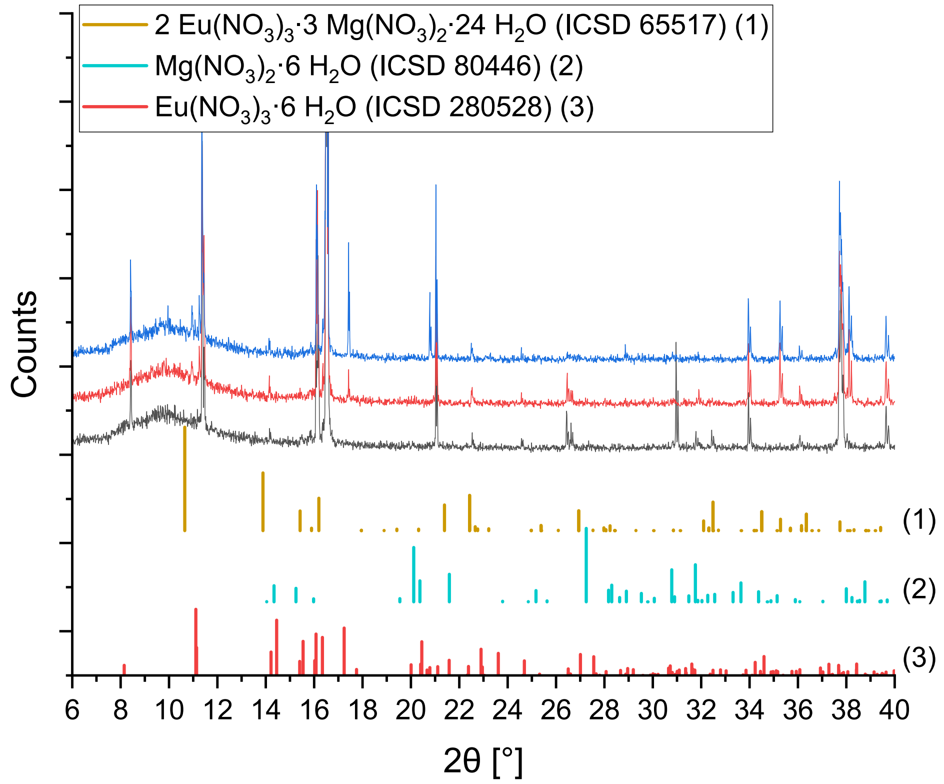

Figure SI- 1: X-ray powder diffraction patterns of sample L (no washing step) in the system Eu(III)-Mg-NO_3-H_2O compared to reference lines[1–3]; consecutive measurements of the same sample with 15 min per measurement in the order black-red-blue (bottom to top).

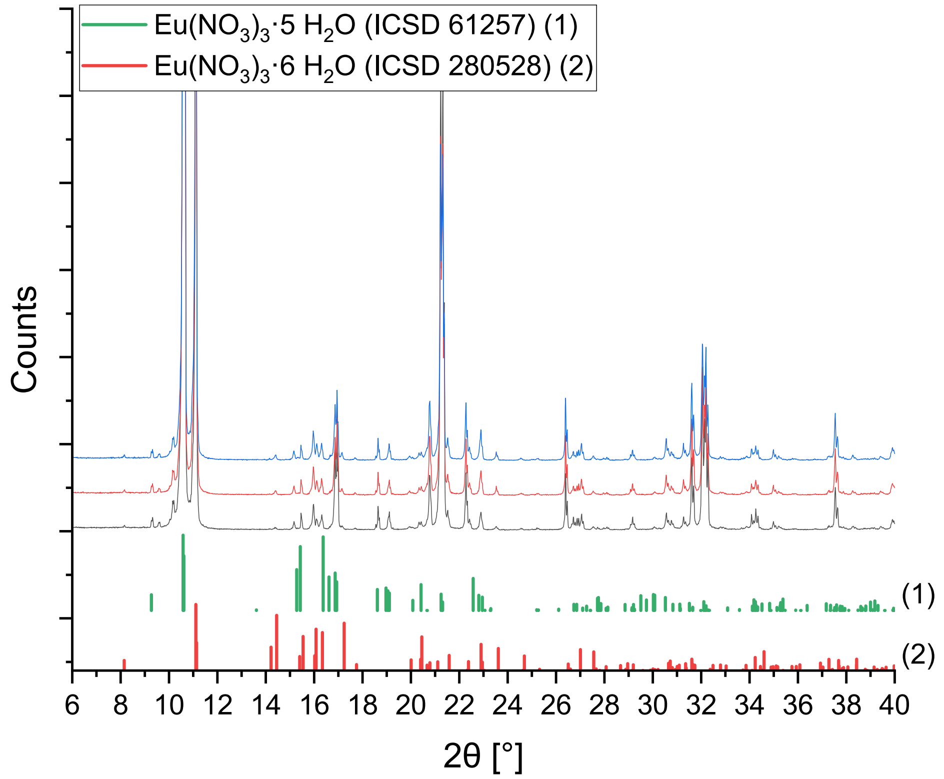

Figure SI- 2: X-ray powder diffraction patterns of sample L (washed with ethanol) in the system Eu(III)-Mg-NO_3-H_2O compared to reference lines[1,4]; consecutive measurements of the same sample with 15 min per measurement in the order black-red-blue (bottom to top).

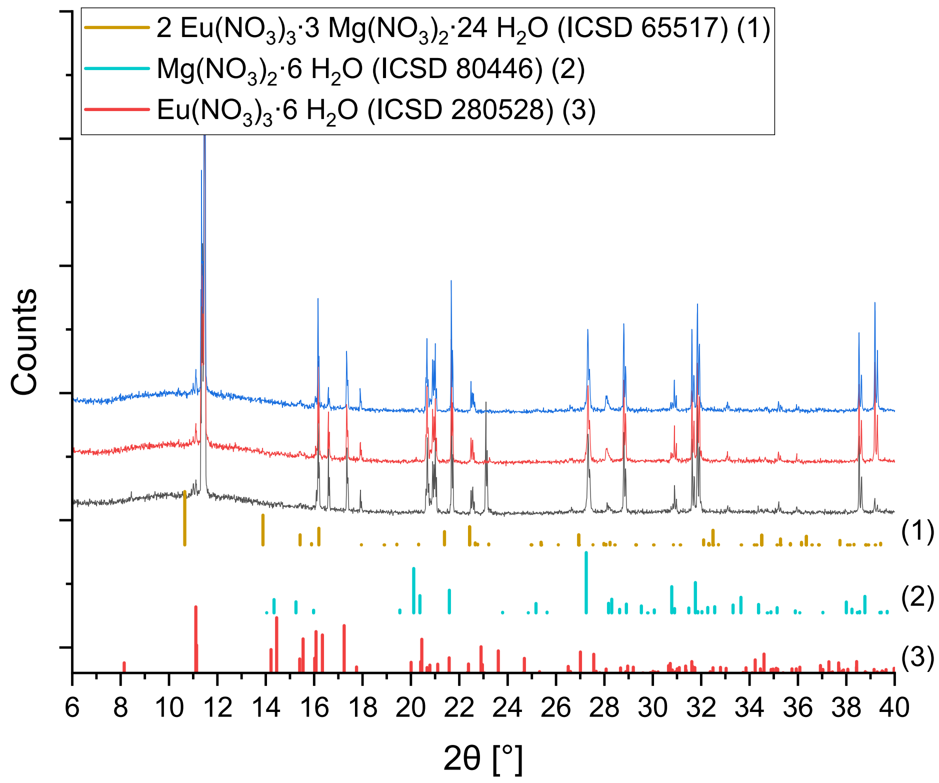

Figure SI- 3: X-ray powder diffraction patterns of sample M (no washing step) in the system Eu(III)-Mg-NO_3-H_2O compared to reference lines[1–3]; consecutive measurements of the same sample with 15 min per measurement in the order black-red-blue (bottom to top).

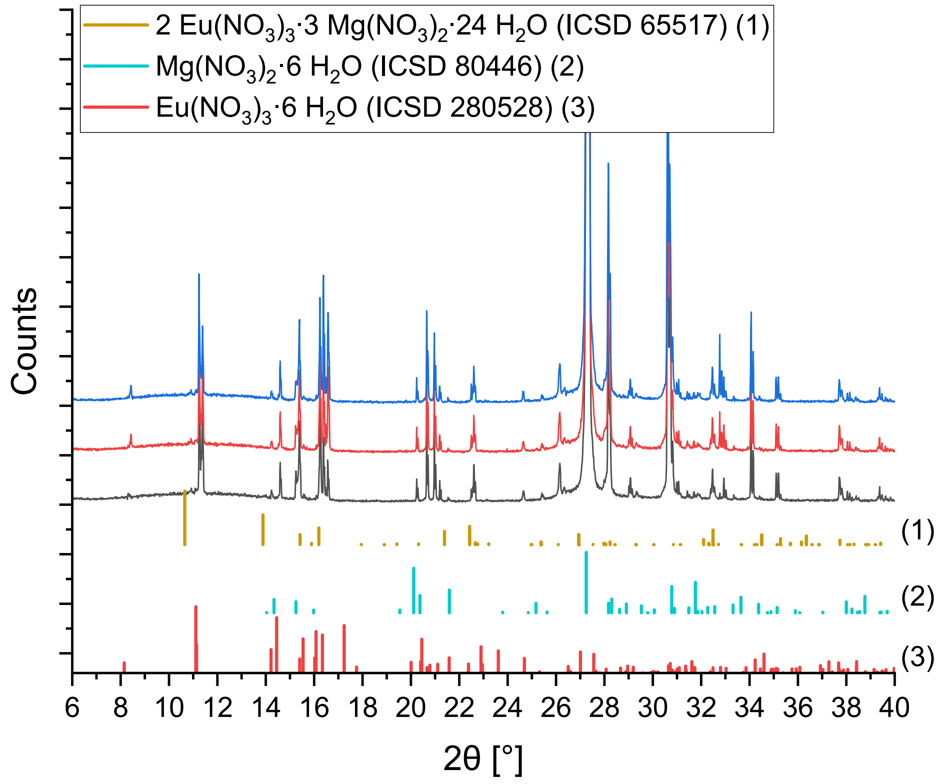

Figure SI- 4: X-ray powder diffraction patterns of sample N (no washing step) in the system Eu(III)-Mg-NO_3-H_2O compared to reference lines[1–3]; consecutive measurements of the same sample with 15 min per measurement in the order black-red-blue (bottom to top).

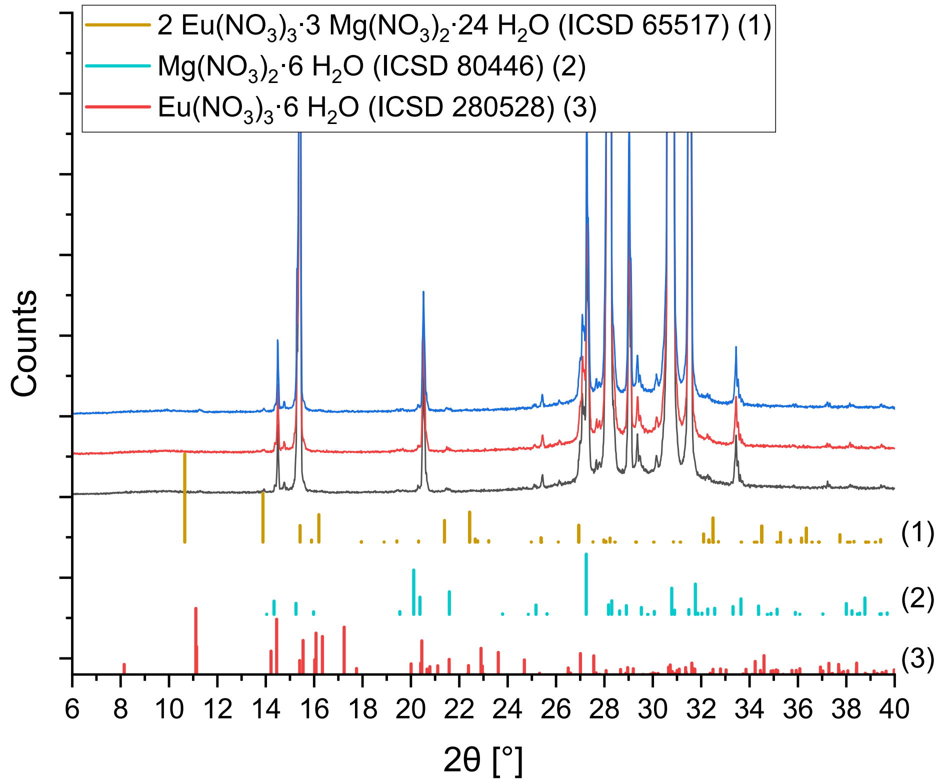

Figure SI- 5: X-ray powder diffraction patterns of sample O (no washing step) in the system Eu(III)-Mg-NO_3-H_2O compared to reference lines[1–3]; consecutive measurements of the same sample with 15 min per measurement in the order black-red-blue (bottom to top).

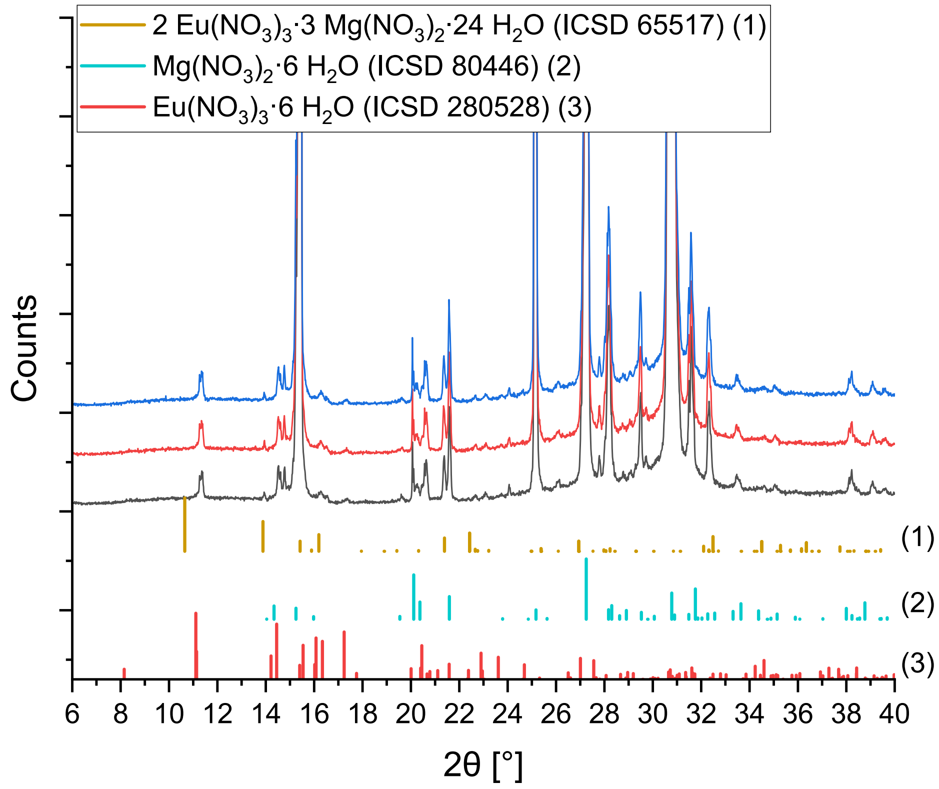

Figure SI- 6: X-ray powder diffraction patterns of sample P (no washing step) in the system Eu(III)-Mg-NO_3-H_2O compared to reference lines[1–3]; consecutive measurements of the same sample with 15 min per measurement in the order black-red-blue (bottom to top).

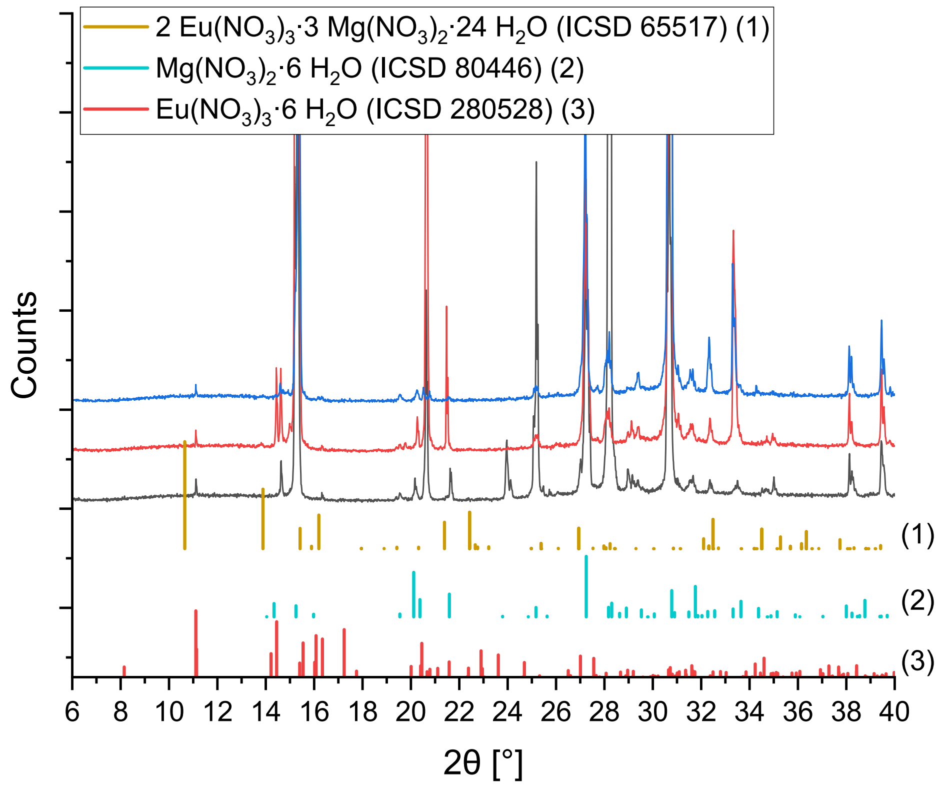

Figure SI- 7: X-ray powder diffraction patterns of sample Q (no washing step) in the system Eu(III)-Mg-NO_3-H_2O compared to reference lines[1–3]; consecutive measurements of the same sample with 15 min per measurement in the order black-red-blue (bottom to top).

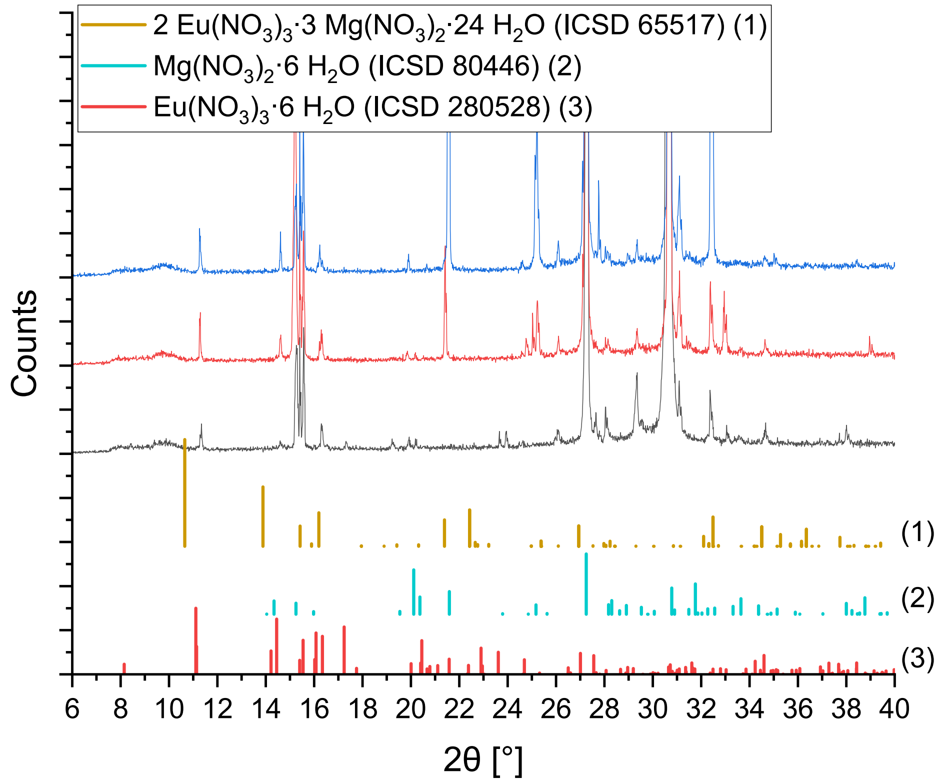

Figure SI- 8: X-ray powder diffraction patterns of sample R (no washing step) in the system Eu(III)-Mg-NO_3-H_2O compared to reference lines[1–3]; consecutive measurements of the same sample with 15 min per measurement in the order black-red-blue (bottom to top).

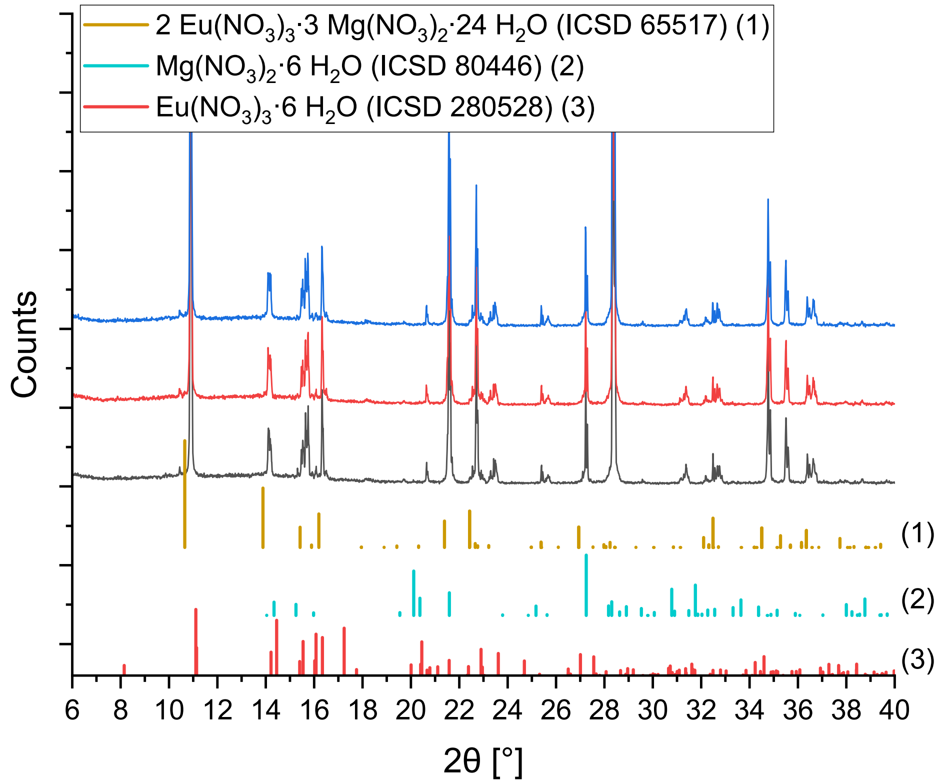

Figure SI- 9: X-ray powder diffraction patterns of sample K (no washing step) in the system Eu(III)-Mg-NO_3-H_2O compared to reference lines[1–3]; consecutive measurements of the same sample with 15 min per measurement in the order black-red-blue (bottom to top).

Rietveld refinement results

Table SI-1: Refined lattice parameters and domain size (coherent scattering domains) of $Eu(NO_3)_3(H_2O)_6$.

Sample	Latice parameters (Å, °) (e.s.d.)						Domain size
	a(Å)	b(Å)	c(Å)	α(°)	β(°)	γ(°)	LVol-IB (nm)
**ICSD 280528**	6.705(1)	9.140(1)	11.647(1)	69.71(1)	88.94(1)	69.29(1)	
**A**	6.7400(2)	9.1530(2)	11.6679(4)	70.071(3)	88.849(3)	69.179(3)	830(90)
**B**	6.7405(3)	9.1522(2)	11.6684(2)	70.021(1)	88.911(3)	69.269(3)	366(8)
**C**	6.707(1)	9.092(1)	11.674(1)	70.121(7)	88.91(1)	69.36(1)	148(7)
**D**	6.7466(4)	9.0688(4)	11.6521(3)	70.121(2)	89.103(2)	69.498(3)	650(110)
**G**	6.7574(3)	9.1786(4)	11.6595(4)	70.039(3)	88.853(3)	69.586(4)	350(40)
**H**	6.7405(4)	9.1521(3)	11.6766(5)	70.020(4)	88.936(5)	69.351(5)	380(50)
**I**	6.7587(3)	9.1413(5)	11.6626(6)	69.873(4)	89.091(3)	69.347(4)	490(160)
**J**	6.7587(4)	9.1419(6)	11.6646(1)	69.860(4)	89.10(1)	69.351(4)	470(90)

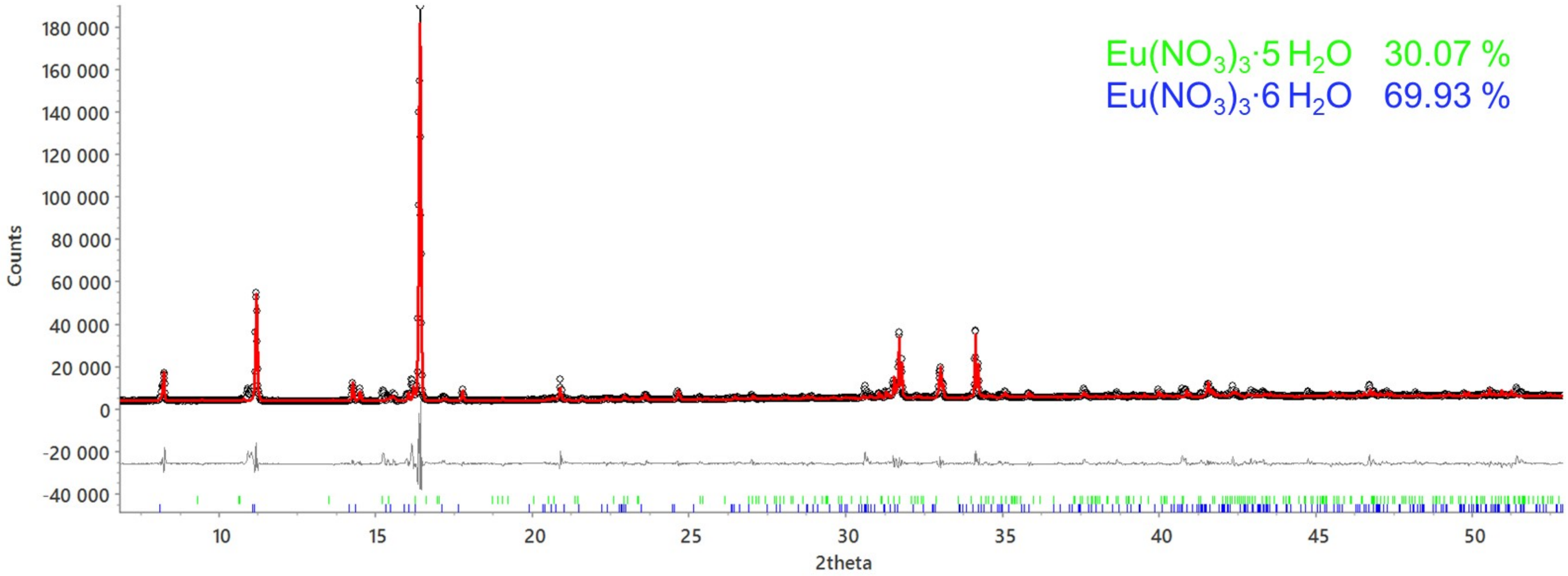

Figure SI- 10: Rietveld plot of XRD sample A; black circles: measured XRD pattern, red line: calculated XRD pattern, grey line: calculated difference between measured and calculated patterns, green and blue: reflection positions of the considered phases.

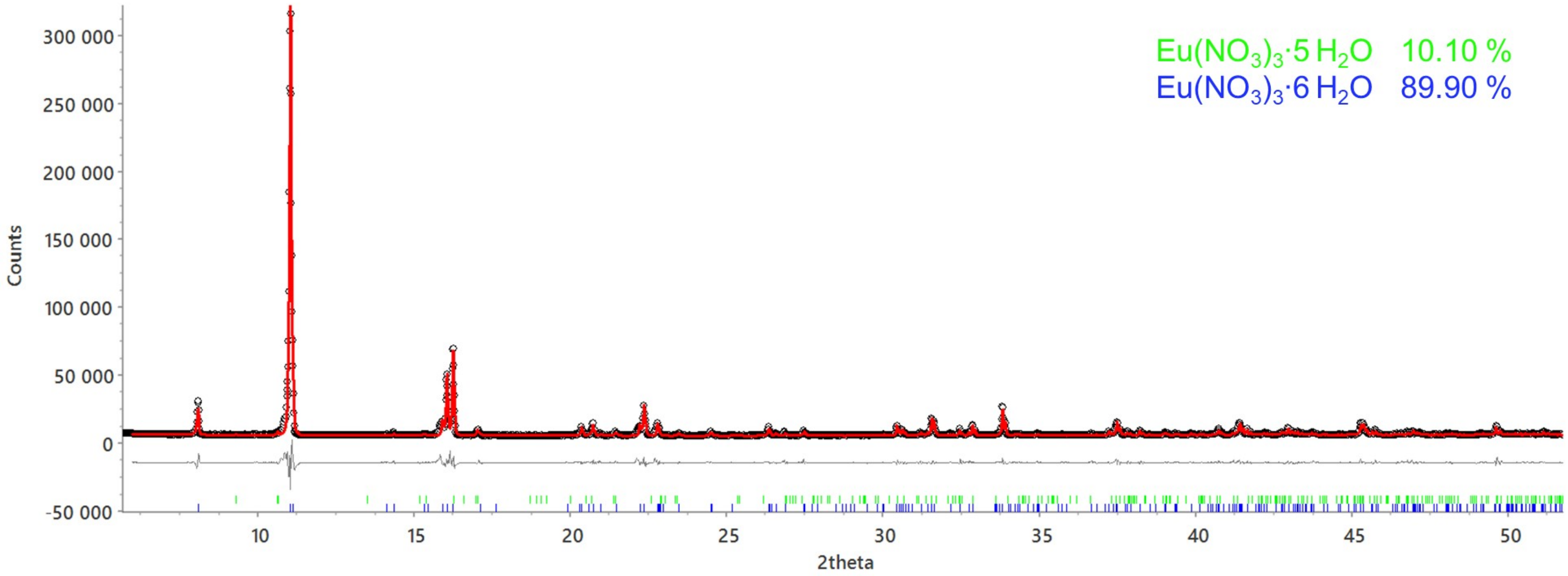

Figure SI- 11: Rietveld plot of XRD sample B; black circles: measured XRD pattern, red line: calculated XRD pattern, grey line: calculated difference between measured and calculated patterns, green and blue: reflection positions of the considered phases.

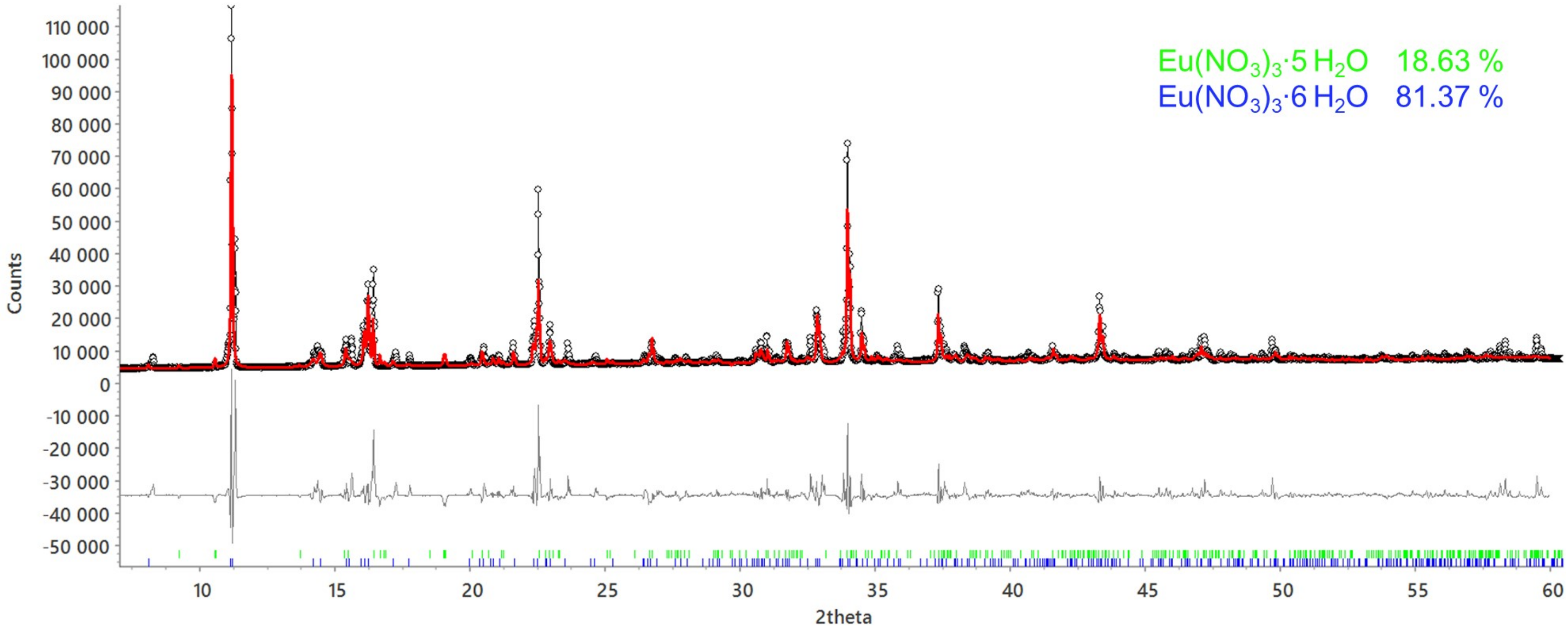

Figure SI- 12: Rietveld plot of XRD sample C; black circles: measured XRD pattern, red line: calculated XRD pattern, grey line: calculated difference between measured and calculated patterns, green and blue: reflection positions of the considered phases.

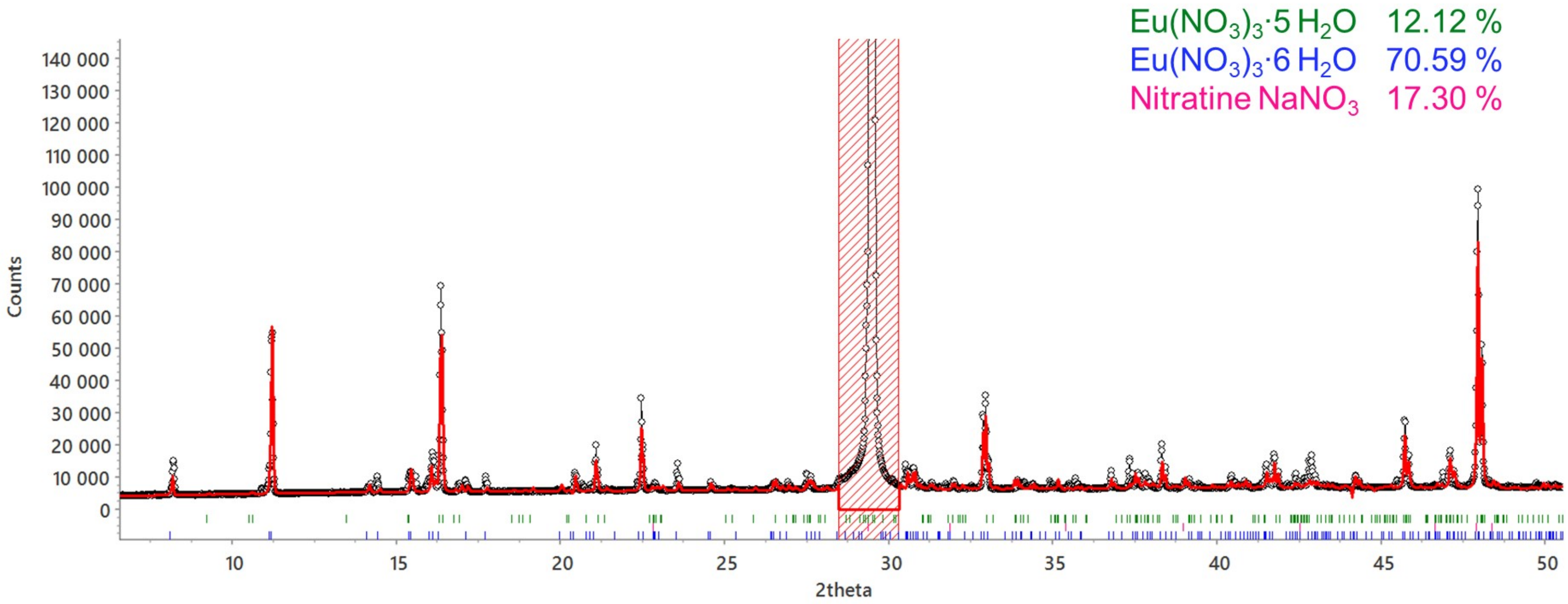

Figure SI- 13: Rietveld plot of XRD sample D; black circles: measured XRD pattern, red line: calculated XRD pattern, grey line: calculated difference between measured and calculated patterns, green, magenta and blue: reflection positions of the considered phases; exclusion of the 104-signal at 29.4 °2θ due to its very strong intensity for a more reasonable quantification of sample.

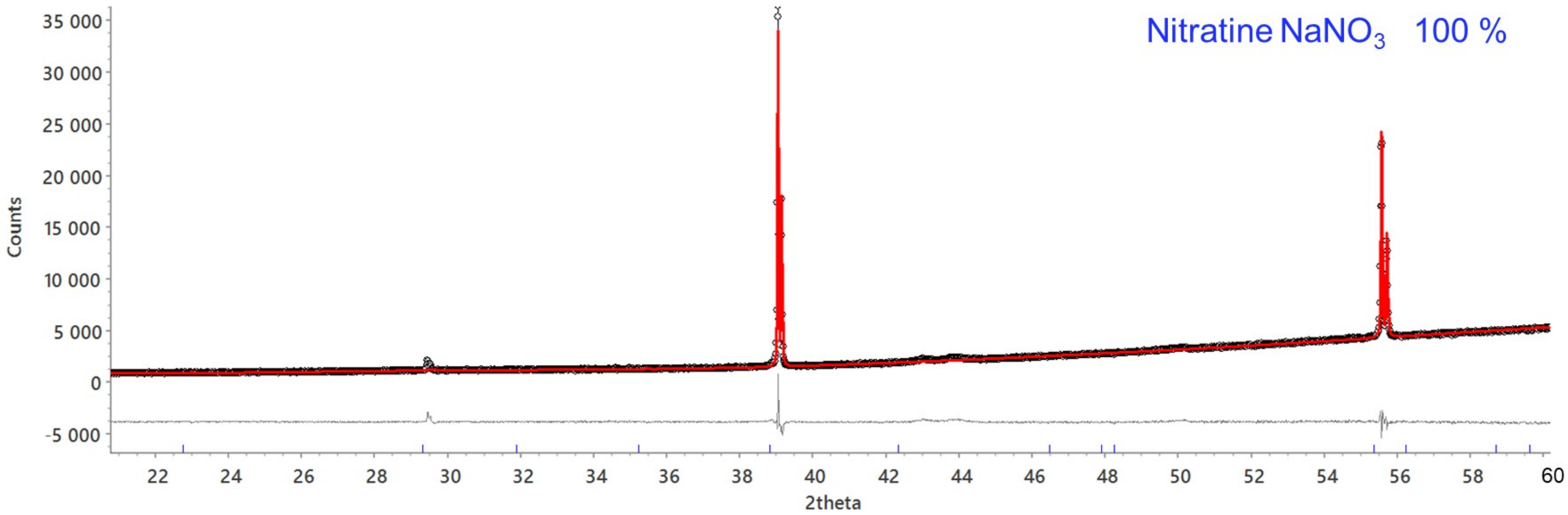

Figure SI- 14: Rietveld plot of XRD sample E; black circles: measured XRD pattern, red line: calculated XRD pattern, grey line: calculated difference between measured and calculated patterns, blue: reflection positions of the considered phase; strong orientation along 2-1-3, 3-1-1, 3-1-5, and 3-1-8 signals of Nitratine in this measurement, refinement of the preferred orientation with spherical harmonic functions is not possible.

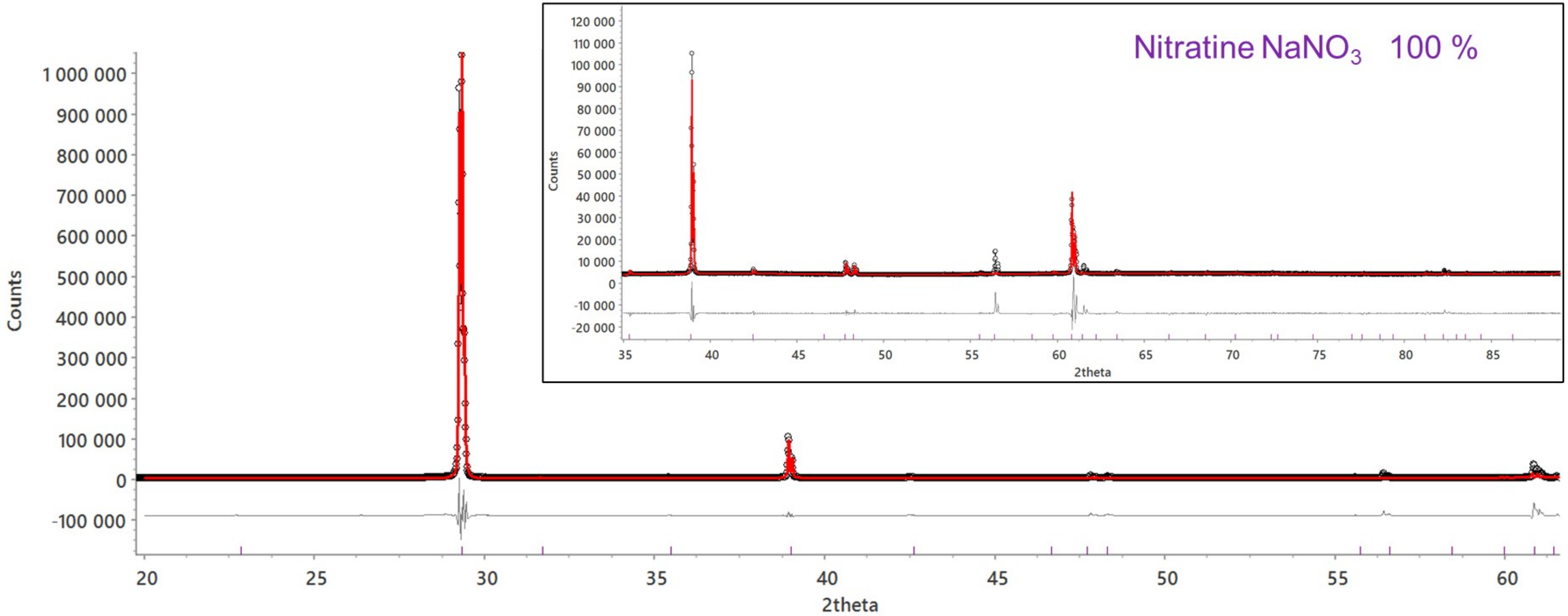

Figure SI- 15: Rietveld plot of XRD sample F; black circles: measured XRD pattern, red line: calculated XRD pattern, grey line: calculated difference between measured and calculated patterns, violet: reflection positions of the considered phase; insert: enlarged presentation of the range 35-90 °2θ.

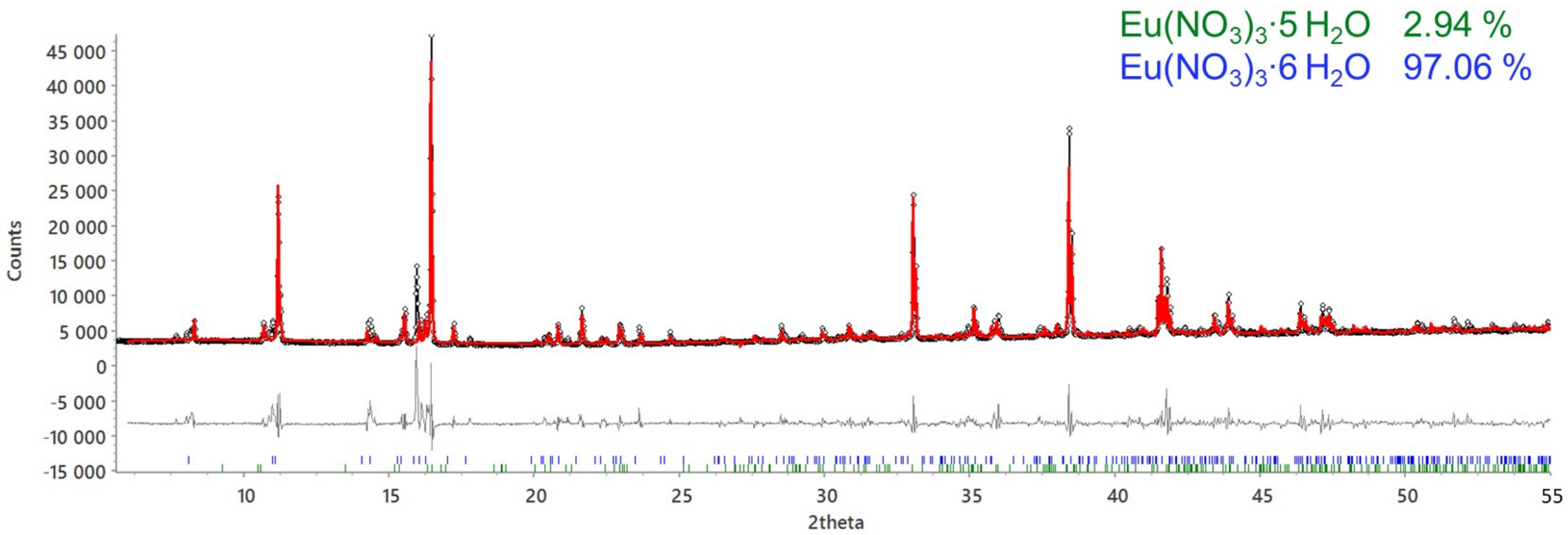

Figure SI- 16: Rietveld plot of XRD sample G; black circles: measured XRD pattern, red line: calculated XRD pattern, grey line: calculated difference between measured and calculated patterns, blue and green: reflection positions of the considered phases.

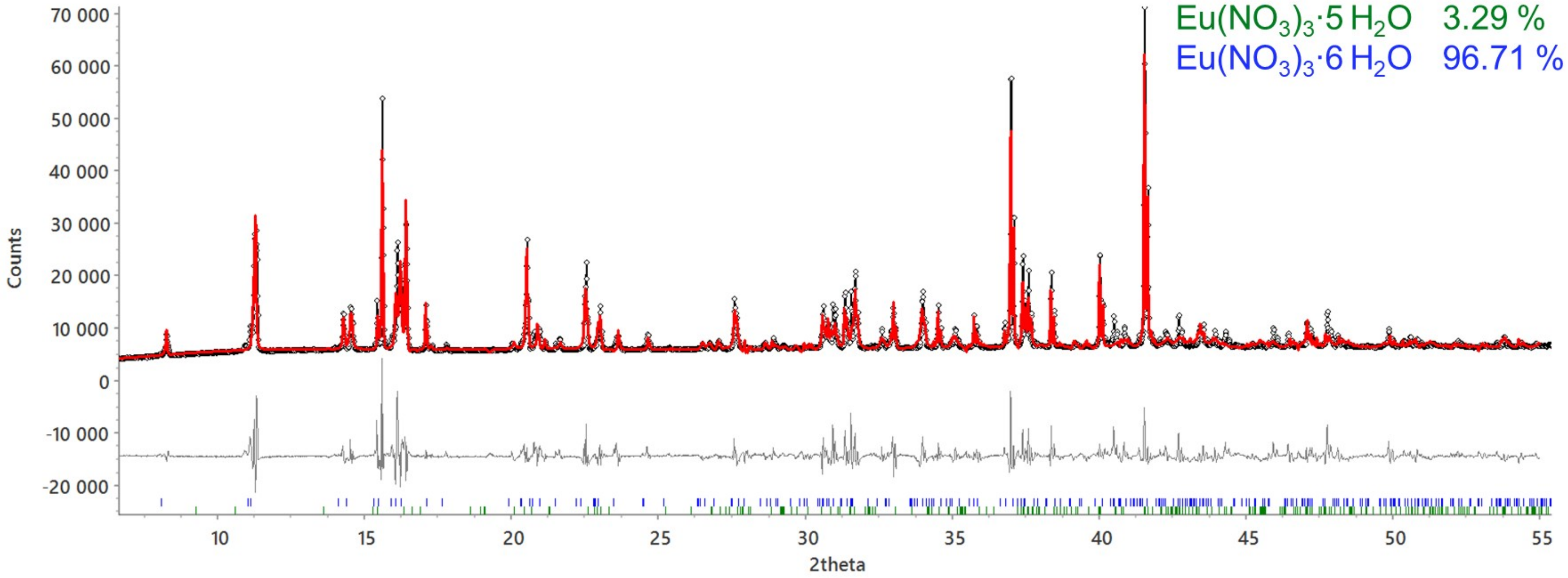

Figure SI- 17: Rietveld plot of XRD sample H; black circles: measured XRD pattern, red line: calculated XRD pattern, grey line: calculated difference between measured and calculated patterns, blue and green: reflection positions of the considered phases.

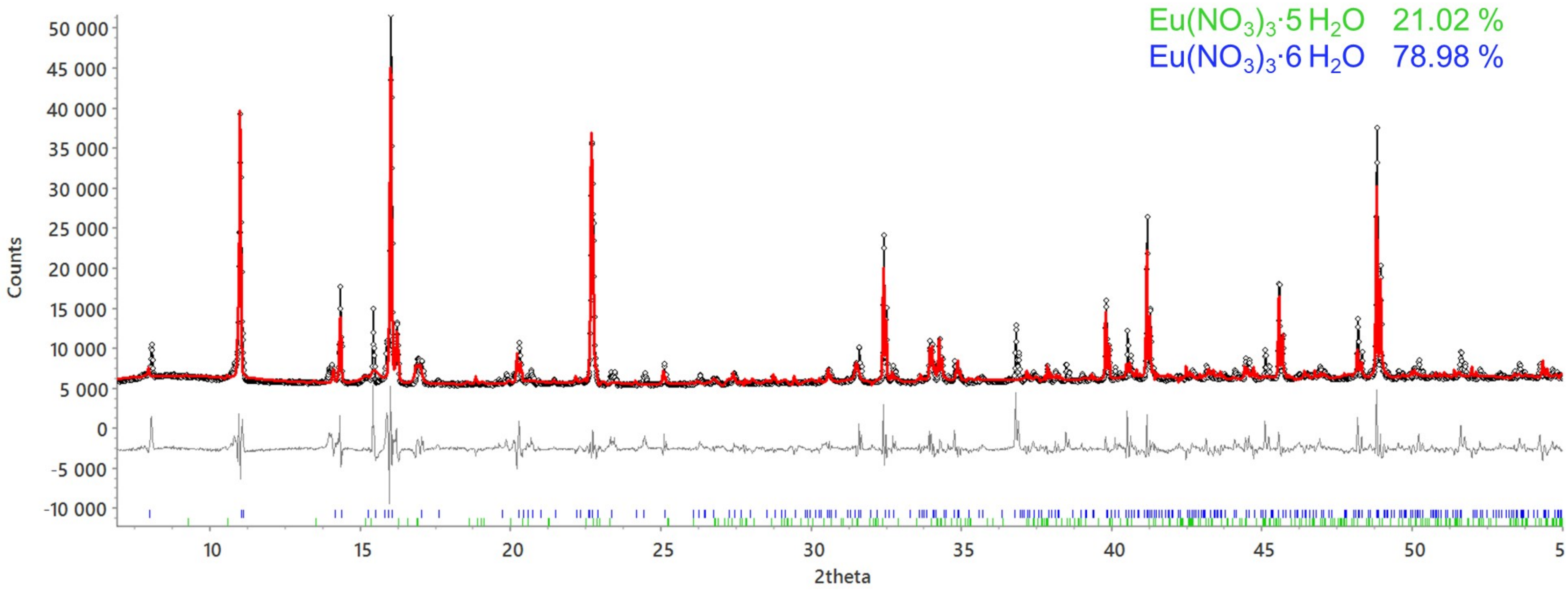

Figure SI- 18: Rietveld plot of XRD sample I; black circles: measured XRD pattern, red line: calculated XRD pattern, grey line: calculated difference between measured and calculated patterns, blue and green: reflection positions of the considered phases.

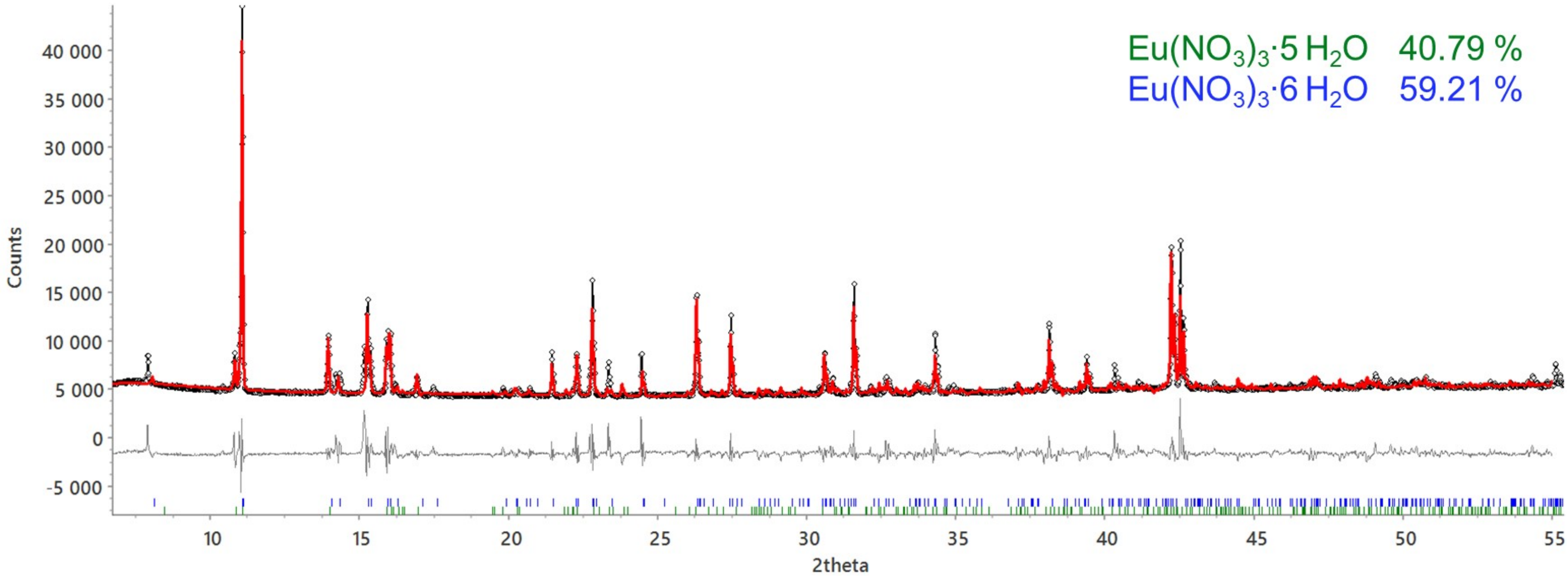

Figure SI- 19: Rietveld plot of XRD sample J; black circles: measured XRD pattern, red line: calculated XRD pattern, grey line: calculated difference between measured and calculated patterns, blue and green: reflection positions of the considered phases.

Comparison of the full dissociation model with osmotic data of Zalupski et al.

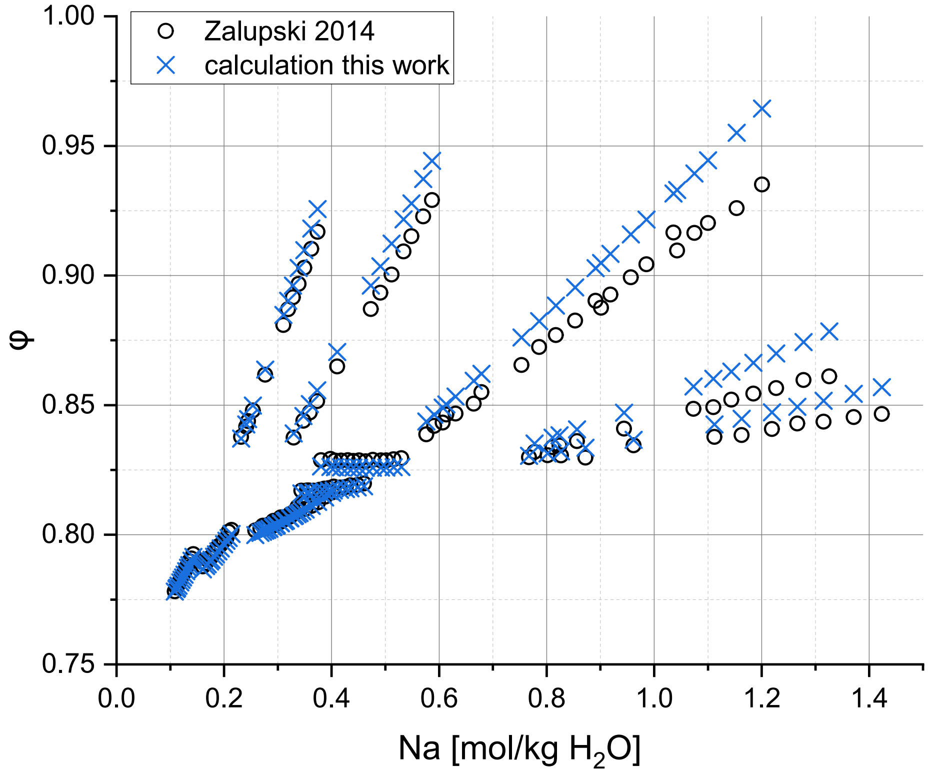

Figure SI- 20: Osmotic data set of Zalupski et al.[5] (black circles) compared to the calculation with the full dissociation Pitzer model of the present work (blue crosses).

References

(1) Stumpf, T.; Bolte, M. Tetra-aqua-trinitratoeuropium(III) Dihydrate. *Acta Cryst E* **2001**, *57* (2), i10–i11. https://doi.org/10.1107/S1600536801001271.

(2) Schefer, J.; Grube, M. Low Temperature Structure of Magnesium Nitrate Hexahydrate, Mg (NO3)2·6H2O: A Neutron Diffraction Study at 173 K. *Materials Research Bulletin* **1995**, *30* (10), 1235–1241. https://doi.org/10.1016/0025-5408(95)00122-0.

(3) Akimov, V. M.; Yanovskii, A. I.; Struchkov, Y. T.; Molodkin, A. K.; Grigor'ev, Y. A.; Novikov, N. K. The Crystal Structure of Magnesium Europium Nitrate-24-Water Mg3Eu2 (NO3) 12 (H2O) 24. *Zh Neorg Khim* **1987**, *32*, 1547–1552.

(4) Ribár, B.; Kapor, A.; Argay, G.; Kálmán, A. Tetraaquatrinitratoeuropium(III) Monohydrate. *Acta Cryst C* **1986**, *42* (10), 1450–1452. https://doi.org/10.1107/S0108270186091928.

(5) Zalupski, P. R.; McDowell, R.; Clegg, S. L. Isopiestic Determination of the Osmotic Coefficients of NaNO3 + Eu(NO3)3 + H2O at 298.15 K and Representation with an Extended Ion-Interaction (Pitzer) Model. *J. Chem. Eng. Data* **2014**, *59* (5), 1574–1582. https://doi.org/10.1021/je500016d.
